# TCP3 is a substrate of the COP1/SPA ubiquitin ligase to regulate anthocyanin accumulation and flowering time in *Arabidopsis*

**DOI:** 10.1073/pnas.2426423122

**Published:** 2025-05-13

**Authors:** Ruiyan Tao, Ira Trivedi, Laura Trimborn, Jathish Ponnu, Blanka Violetta Tóth, Ute Hoecker

**Affiliations:** ^a^Institute for Plant Sciences and Cluster of Excellence on Plant Sciences (CEPLAS), Department of Biology, Biocenter, University of Cologne, Cologne 50674, Germany

**Keywords:** COP1/SPA complex, ubiquitin ligase, TCP transcription factor family, TCP3, flowering time

## Abstract

COP1 is a conserved ubiquitin ligase found in plants and animals. In plants, COP1 acts together with SPA proteins to suppress light signaling in darkness by promoting the degradation of transcription factors involved in photomorphogenesis. Substrates of this ubiquitin ligase share a Valine-Proline (VP) motif that interacts with the WD-repeat domain of COP1 in plants and humans. Here, we have identified the transcription factor TCP3 as a noncanonical substrate of COP1/SPA that lacks a VP motif. The TCP domain of TCP3 directly interacts with the WD-repeat domains of COP1 and SPA1. TCP3 requires the VP-binding cleft of COP1 for protein–protein interaction. We further show that the TCP3 protein is degraded in darkness and preferentially in short day through a COP1-dependent manner, while TCP3 is stabilized by red, far-red, blue light, and long day conditions. COP1/SPA-mediated degradation of TCP3 inhibits anthocyanin accumulation by reducing the expression of anthocyanin biosynthesis genes. COP1/SPA-mediated degradation of TCP3 is also important in regulating flowering time. Taken together, our results have identified a noncanonical substrate of the COP1/SPA ubiquitin ligase, thereby also uncovering TCPs as a transcription factor family that is targeted by COP1/SPA. Since the COP1/SPA-interacting TCP domain is conserved among TCPs, it is possible that other members of the TCP family—having divergent functions including cell fate determination and hormone signaling—are targets of COP1/SPA as well.

## Significance

Light is an important environmental cue that enables plants to adapt to the ambient light conditions. A key component of the light signaling network is the COP1/SPA ubiquitin ligase which causes degradation of multiple transcription factors. These transcription factors share a Valine-Proline (VP) motif responsible for binding COP1/SPA. Here, we have identified a degradation substrate (TCP3) that is recognized by COP1/SPA in a noncanonical, VP-independent fashion. We demonstrate that COP1/SPA promotes TCP3 degradation in darkness and preferentially under short day. This process inhibits the biosynthesis of anthocyanin and delays flowering. Our findings not only expand the substrate selection mode of COP1/SPA, but also uncovered a transcription factor family involved in the complex COP1/SPA-dependent signaling network.

Light has a crucial role in the growth and development of plants. Throughout the entire developmental cycle of plants, light has an essential function in various biological processes, such as seed germination, seedling de-etiolation, anthocyanin biosynthesis, shade avoidance, and flowering time (1). Plants have evolved specialized photoreceptors that sense ambient light conditions. Previous studies have identified different types of photoreceptors, including phytochromes which capture red and far-red light, cryptochromes and phototropins which capture blue and UV-A light and UV resistance locus 8 (UVR8) which captures UV-B (2–4).

Light-activated phytochrome, cryptochrome, and UVR8 photoreceptors bind and inhibit the CULLIN4-based CONSTITUTIVELY PHOTOMORPHOGENIC 1 (COP1)-SUPPRESSOR OF PHYA-105 (SPA1) E3 ubiquitin ligase, thereby allowing photomorphogenesis to proceed (5, 6). The CUL4^COP1-SPA^ complex acts as a repressor of photomorphogenesis, working mostly in darkness or in the shade (7, 8). Most of its substrates are factors that promote photomorphogenesis, including transcription factors such as HY5, CO, and PAP2 (8, 9).

The COP1/SPA complex consists of two COP1 and two SPA proteins from the four-member SPA protein family (SPA1–SPA4) (10, 11). Both COP1 and SPA are essential for the proper function of this complex (12). Structurally, COP1 has three main domains: the RING-finger domain, the coiled-coil domain, and the WD repeat domain. Similarly, SPA proteins contain three structural domains: a kinase domain (KIN domain), the coiled-coil domain, and the C-terminal WD repeats (10, 13). The interaction between COP1 and SPA is dependent on the respective coiled-coil domains (11, 13). The WD repeat domains of COP1 and SPA proteins interact with a VP motif present in many substrates (8, 13, 14, 15). The VP motif was defined as VP(E/D)ΦG (where Φ represents a hydrophobic residue) (14). Crystallography confirmed that this motif is directly involved in binding COP1-WD from *Arabidopsis* and humans, indicating conservation of the COP1-WD–substrate interaction between plants and animals (6, 15). Besides, photoactivated cryptochromes and UVR8 competitively interact with COP1 through their VP motif, thereby weakening the interaction between COP1 and HY5 or PAP2 (6, 16, 17).

TEOSINTE BRANCHED 1/CYCLOIDEA/PCF (TCP) proteins are a group of plant-specific transcription factors, which are characterized by a noncanonical basic helix–loop–helix motif, the TCP domain, which functions as a DNA-binding and dimerization domain (18–22). In *Arabidopsis*, a total of 24 TCPs is categorized into two subclasses based on sequence variation in the TCP domain (22, 23). Subclass II is further subdivided into three TB1/CYC-like TCPs (TCP1, TCP12, and TCP18) and eight CINCINNATA-like TCPs (CIN-TCPs) (TCP2, TCP3, TCP4, TCP5, TCP10, TCP13, TCP17, and TCP24). Five of the CIN-TCPs are targeted by miR319 (24). TCP proteins can be subject to posttranslational modifications including ubiquitination and SUMOylation (25–27). Heterodimerization among TCPs and protein–protein interactions with other transcription factors further enhance diversity of TCP functions (28–30).

TCPs play roles in diverse biological processes, including leaf development, shoot branching, trichome formation, flower morphology, flowering time, hormone signaling, and photomorphogenesis (22). Previous studies showed that TCP3, TCP4, and TCP10 promote photoperiod-induced flowering of *Arabidopsis* by enhancing the expression of *CONSTANS* (*CO*), while TCP7 interacts with Nuclear Factor-Ys and stimulates *SOC1* expression to promote flowering in *Arabidopsis* (31–33). Besides, TCP3 promotes anthocyanin accumulation by interacting with MYB proteins of the R2R3-MYB/bHLH/WD40 (MBW) complex, thereby enhancing MYB–bHLH interaction and expression of anthocyanin biosynthesis genes (34). Heterologous expression of the chimeric TCP3 repressor reduces anthocyanin accumulation in *Torenia fournieri* flowers (35). TCP4 was also reported to increase anthocyanin accumulation under white light and UV-B via regulating JA-response genes (36). In contrast, TCP15 was reported to inhibit anthocyanin accumulation in high light (37). Moreover, TCP4 promotes cotyledon opening by weakening the binding of PIF3 to the *SAURs* promoters, and TCP15 induces *SAUR*, *EXPB1,* and other genes by interacting with GLK1 to promote cotyledon opening (38, 39).

In this study, we identified TCP3 as a COP1/SPA-interacting protein which lacks a canonical VP motif for COP1/SPA interaction. We show that TCP3 is a degradation substrate of the COP1/SPA ubiquitin ligase. Destabilization of TCP3 in darkness and preferentially in short day regulates anthocyanin accumulation and flowering time.

## Results

### TCP3 Physically Interacts with COP1 and SPA1.

**Fig. 1.**
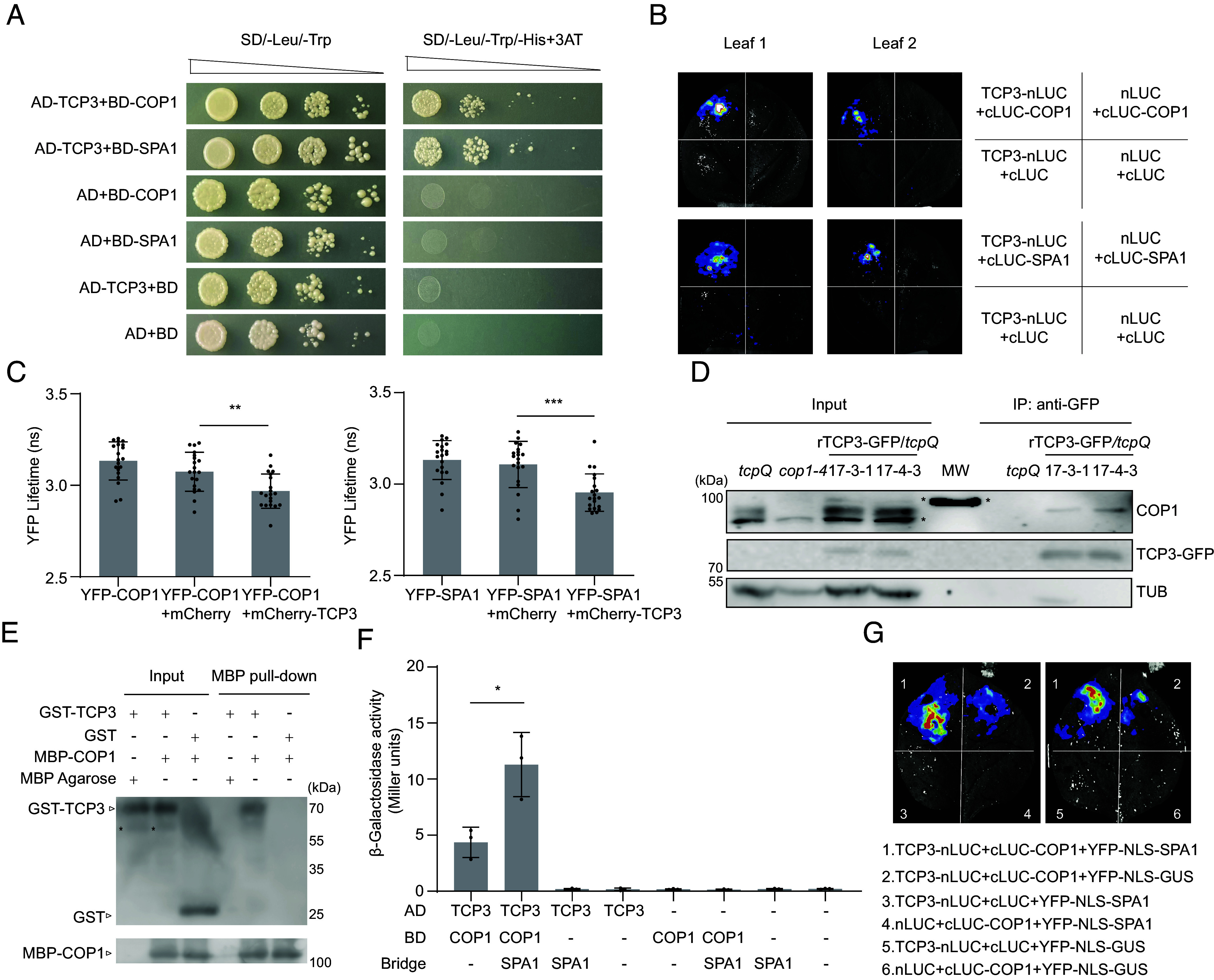
Physical interaction between TCP3 and the COP1–SPA1 complex. (*A*) Yeast two-hybrid (Y2H) assays studying the interaction between TCP3 and COP1 or SPA1. BD-COP1 or BD-SPA1 was constructed as bait, and AD-TCP3 as prey. Transformed yeast cells were grown on the indicated media at decreasing OD. (*B*) LCI assays. SPA1 and COP1 were fused to the C-terminal half of luciferase (cLUC), and TCP3 was fused to the N-terminal half of luciferase (nLUC). Representative images of two tobacco leaves that were transfected with the indicated plasmid combinations. (*C*) FRET-FLIM analysis of leek cells transfected with the indicated plasmid combinations using particle bombardment. Error bars represent SD of 20 analyzed cells. Asterisks indicate significant differences in lifetime of the indicated pairs using Student’s *t* test (**P < 0.01 and ***P < 0.001). (*D*) Coimmunoprecipitation of COP1 and TCP3-GFP in *Arabidopsis* seedlings. Seedlings of the indicated genotypes were grown under continuous white light (Wc, 100 μmol m^−2^ s^−1^) for 7 d, treated with 50 μM MG132 and then shifted to darkness for 4 h. TCP3-GFP was immunoprecipitated using GFP beads, and coprecipitated COP1 was detected with an α-COP1 antibody. TCP3-GFP was identified using an α-GFP antibody. Tubulin (TUB) served as a loading control for input samples, detected by anti-α-tubulin antibody. Samples were separated on two gels: one for GFP and TUB detection and another one for COP1 detection. MW = molecular weight marker. Asterisks indicate nonspecific signals detected in the plant extracts and also in the MW. (*E*) Pull-down assays between GST-TCP3 and MBP-COP1. The GST-TCP3, GST, and MBP-COP1 proteins were purified from *Escherichia coli*. The experimental setup included combinations of GST-TCP3, GST-TCP3 + MBP-COP1, and GST + MBP-COP1, which were coprecipitated using MBP-beads and subsequently eluted. The eluates were analyzed using α-GST and α-MBP antibodies. (*F*) SPA1 enhances the COP1–TCP3 complex formation in Y3H assay. BD-COP1 as bait and AD-TCP3 as prey were expressed in the presence or absence of coexpressed, untagged SPA1 (“Bridge”). Error bars represent the SD from three independent biological replicates. The asterisk indicates a significant difference between the indicated pairs (Student’s *t* test, *P < 0.05). (*G*) SPA1 enhances the COP1–TCP3 complex formation in LCI assays. nLUC-COP1, TCP3-cLUC, and respective negative controls were coexpressed with YFP-NLS-tagged SPA1 or YFP-NLS-tagged GUS (negative control) in tobacco leaves. Representative images of two tobacco leaves transfected with the indicated construct combinations are shown. Quantification of luciferase activities and YFP fluorescence are shown in *SI Appendix*, Fig. S2.

We used a yeast two-hybrid (Y2H) transcription factor library (40, 41) to screen for transcription factors that interact with COP1 and/or SPA1. This screen identified TCP3 as a COP1- and SPA1-binding protein. We validated the interactions in a directed Y2H assay: AD-TCP3 engaged in protein–protein interactions with both BD-COP1 and BD-SPA1 (Fig. 1*A*). Subsequently, we confirmed these protein–protein interactions in plants. Tobacco-based luciferase complementation imaging (LCI) revealed that expression of nLUC-COP1 or nLUC-SPA1 with TCP3-cLUC produced obvious luminescence, whereas the negative controls displayed no or only minimal luminescence (Fig. 1*B*). In colocalization experiments using bombarded leek epidermal cells, YFP-COP1 and YFP-SPA1 formed nuclear bodies that colocalized with mCherry-TCP3 (*SI Appendix*, Fig. S1). mCherry-TCP3 did not form nuclear bodies in the absence of coexpressed COP1 or SPA1 (*SI Appendix*, Fig. S1). This indicates that COP1 and SPA1 recruited TCP3 into their nuclear bodies. Using these transfected leek epidermal cells, we also performed Förster resonance energy transfer-fluorescence lifetime imaging microscopy (FRET-FLIM). These results show that coexpression of mCherry-TCP3 with YFP-COP1 resulted in a reduction in the lifetime of the donor fluorophore (YFP) compared to the coexpression of YFP-COP1 and mCherry. Similarly, YFP-SPA1 and mCherry-TCP3 coexpression led to a reduction in YFP fluorescence lifetime compared to the negative control (Fig. 1*C*).

To confirm the TCP3–COP1 interaction in *Arabidopsis* seedlings, we performed coimmunoprecipitation assays. To this end, we generated stable transgenic lines expressing GFP-tagged TCP3. Since miR319 down-regulates *TCP3* at the mRNA level (24, 42), we altered the DNA sequence of the miR319-binding site in *TCP3* while keeping the encoded amino acid sequence unchanged, naming this *rTCP3* for miRNA-resistant *TCP3* (*SI Appendix*, Fig. S2 *A* and *B*). We transformed *35S::rTCP3-GFP* into the *tcp2tcp3tcp4tcp10* quadruple mutant (*tcpQ*) background because *tcp3* single mutants do not show a mutant phenotype due to functional redundancy among CIN-TCPs (43). The *rTCP3-GFP* transgene rescued the *tcpQ* mutant phenotype (see later), indicating that the transgene is functional. In two independent homozygous rTCP3-GFP/*tcpQ* lines (17-3-1 and 17-4-3), rTCP3-GFP successfully coimmunoprecipitated endogenous COP1, whereas nontransgenic *tcpQ* seedlings did not copurify COP1 (Fig. 1*D*).

To further confirm that the interaction between TCP3 and COP1 is a direct protein–protein interaction rather than an indirect one, we purified GST-TCP3 and MBP-COP1 from *E. coli* and performed an in vitro pull-down assay. The results show that MBP-COP1 was able to pull down GST-TCP3 but not GST, indicating a direct protein–protein interaction between GST-TCP3 and MBP-COP1 (Fig. 1*E*).

Since COP1 and SPA1 function as a complex, we hypothesized that SPA1 might enhance the complex formation between COP1 and TCP3. To test this hypothesis, we conducted yeast three-hybrid experiments by coexpressing SPA1 with BD-COP1 and AD-TCP3 and LCI assays by coexpressing YFP-NLS-SPA1 or YFP-NLS-GUS with nLUC-COP1 and TCP3-cLUC. The results indicate that the presence of SPA1 enhanced the β-galactosidase activity between BD-COP1 and AD-TCP3 in yeast and luminescence intensity between nLUC-COP1 and TCP3-cLUC in tobacco (Fig. 1 *F* and *G* and *SI Appendix*, Fig. S3). In summary, these findings suggest that SPA1 enhances the complex formation between COP1 and TCP3, likely by serving as a bridge protein between COP1 and TCP3 and providing an additional TCP3-interacting WD-repeat domain.

Taken together, these experiments demonstrate that TCP3 interacts with COP1 and SPA1 in vivo and in vitro.

### COP1 and SPA1 Exhibit Partially Distinct Interaction Domains with TCP3.

To define the interacting domains in COP1, SPA1, and TCP3, we tested deletion-derivatives of these proteins. We divided COP1 into its N-terminal half (COP1-N) comprising the RING-finger (RF) and coiled-coil (CC) domains, and its C-terminal half (COP1-C) containing the WD domain. Similarly, SPA1 was divided into its N-terminal half (SPA1-N) which includes the kinase (KIN) and CC domains, and its C-terminal half (SPA1-C) which contains the WD domain (*SI Appendix*, Fig. S4 *A* and *C*). In Y2H assays, both BD-COP1-C and BD-SPA1-C interacted with AD-TCP3 (*SI Appendix*, Fig. S4 *B* and *D*). Additionally, we observed a protein–protein interaction between the BD-SPA1-N and AD-TCP3 (*SI Appendix*, Fig. S4*D*). This experiment demonstrates that COP1 relies on its WD-repeat domain to interact with TCP3, whereas SPA1 can interact with TCP3 through both its WD-repeat domain and its N-terminal domains.

### TCP3, TCP4, and TCP10 Interact with COP1 Independently of a VP Motif.

**Fig. 2.**
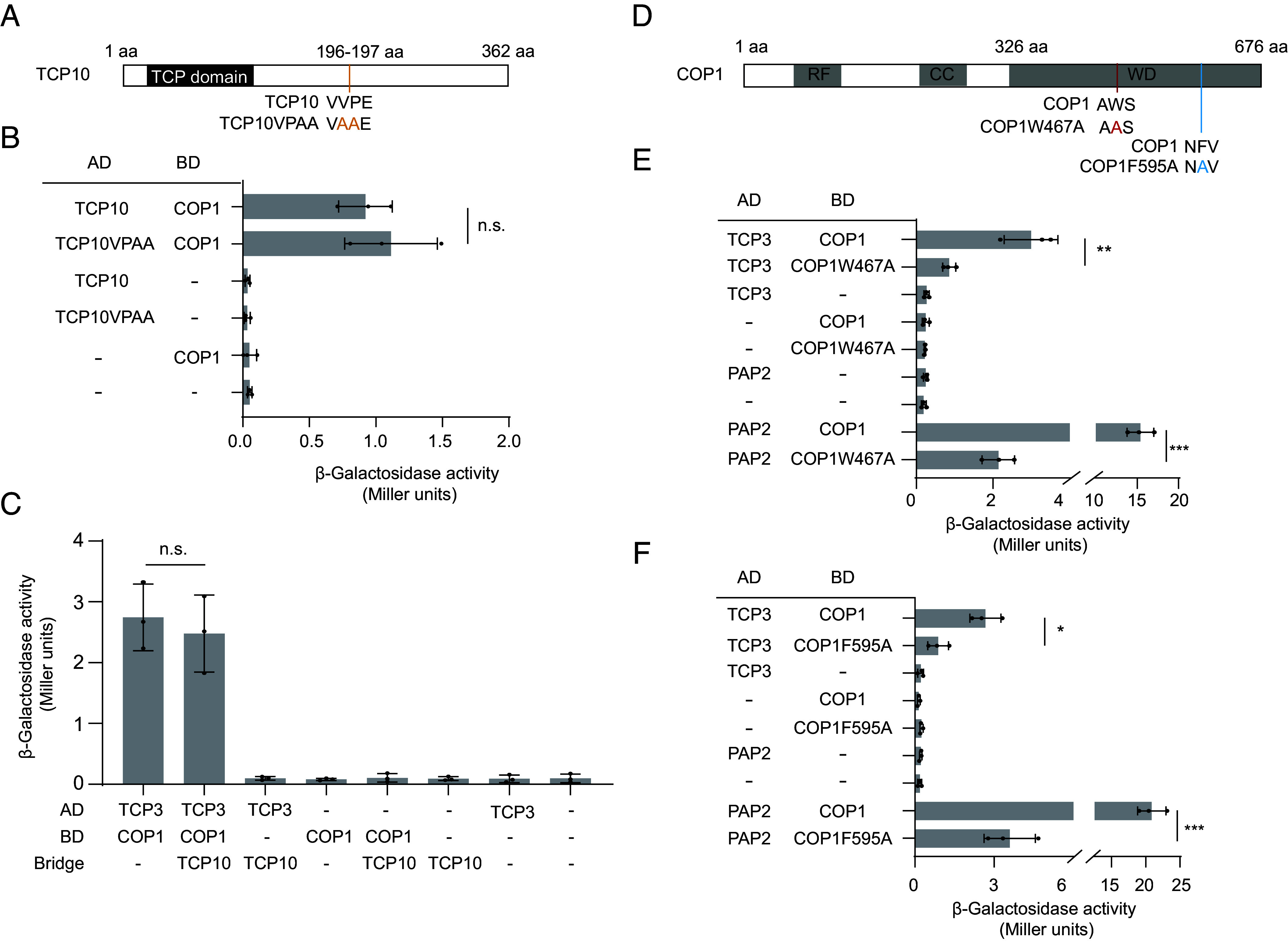
TCP3 and TCP10 interact with COP1 independently of a VP motif. (*A* and *B*) Y2H assays studying the interaction between AD-TCP10 or AD-TCP10VPAA and BD-COP1. The schematic diagram shows the mutation sites of TCP10VPAA (*A*). Error bars represent the SD from three independent biological replicates (*B*). (*C*) TCP10 fails to affect the protein–protein interaction between COP1 and TCP3. BD-COP1 and AD-TCP3 were expressed in the presence or absence of coexpressed, untagged TCP10 (Bridge). Error bars represent the SD from three independent biological replicates. (*D*–*F*) Y2H assays studying the interaction between TCP3 and two COP1 mutants. Schematic diagram showing the mutations in COP1 (*D*). The interaction between BD-COP1W467A and AD-TCP3 is shown in *E*, while the interaction between BD-COP1F595A and AD-TCP3 is shown in *F*. In both experiments, AD-PAP2 was used as a positive control for a VP-containing interactor. Error bars represent the SD from three independent biological replicates. Asterisks in *B*, *C*, *E*, and *F* denote significant differences between the indicated pairs (Student’s *t* test, ***P < 0.001 **P < 0.01, *P < 0.05, n.s. = not significant).

TCP3 belongs to the TCP protein family, with TCP4 and TCP10 closely related to TCP3. Indeed, TCP4 and TCP10 also bound COP1 and SPA1 in Y2H (*SI Appendix*, Fig. S4*E*). Interestingly, TCP3 and TCP4 lack a canonical VP motif (*SI Appendix*, Fig. S2*C*) which is present in most COP1/SPA targets (8, 14, 15). In contrast, TCP10 may contain a VP motif (*SI Appendix*, Fig. S2*C*). However, when the VP domain of TCP10 was mutated, its interaction with COP1 remained intact (Fig. 2 *A* and *B*). Additionally, since TCPs often function as heterodimers, we aimed to determine whether the VP-containing TCP10 could promote the interaction between TCP3 and COP1. Results from Y3H assays indicate that TCP10 did not affect the protein–protein interaction between TCP3 and COP1 (Fig. 2*C*). These observations indicate that TCP3, TCP4, and TCP10 are noncanonical interactors of COP1-WD because they lack a canonical VP motif. Since the interaction between COP1 and VP-containing substrates typically relies on the VP-binding cleft of the COP1 WD domain (6, 14, 15), we were curious whether TCP3, which lacks a VP motif, also binds to this region. Based on previous studies, we constructed two COP1 mutants, COP1W467A and COP1F595A, which disrupt the VP-binding pocket within the COP1 WD domain (Fig. 2*D*) (6, 14). Y2H assays revealed that the interaction between TCP3 and COP1 was strongly reduced when either site was mutated, and this reduction was similar to the one observed for the positive control, the VP-containing substrate PAP2 (Fig. 2 *E* and *F*). These findings suggest that, despite lacking a VP domain, TCP3 still interacts with the VP-binding pocket of COP1.

### The TCP Domain of TCP3 Is Responsible for Interacting with COP1 and SPA1.

To identify the regions of TCP3 responsible for its interaction with COP1 and SPA1, we divided TCP3 into four segments (*SI Appendix*, Fig. S5*A*). FRET-FLIM assays showed that coexpression of mCherry-TCP3-D1 or mCherry-TCP3-D3 reduced the lifetime of YFP-COP1 and YFP-SPA1 (*SI Appendix*, Fig. S5*B*). Similarly, colocalization assays confirmed these results: mCherry-TCP3-D1 and mCherry-TCP3-D3 colocalized with YFP-COP1 and YFP-SPA1 (*SI Appendix*, Fig. S5*C*). In Y2H assays, both full-length BD-COP1 and BD-SPA1 interacted with AD-TCP3-D1 and AD-TCP3-D3 (*SI Appendix*, Fig. S5*D*). Taken together, these results indicate that COP1 and SPA1 interact with the TCP domain of TCP3.

### TCP3 Is Degraded in Darkness and Stabilized By Red, Far-Red, and Blue Light.

**Fig. 3.**
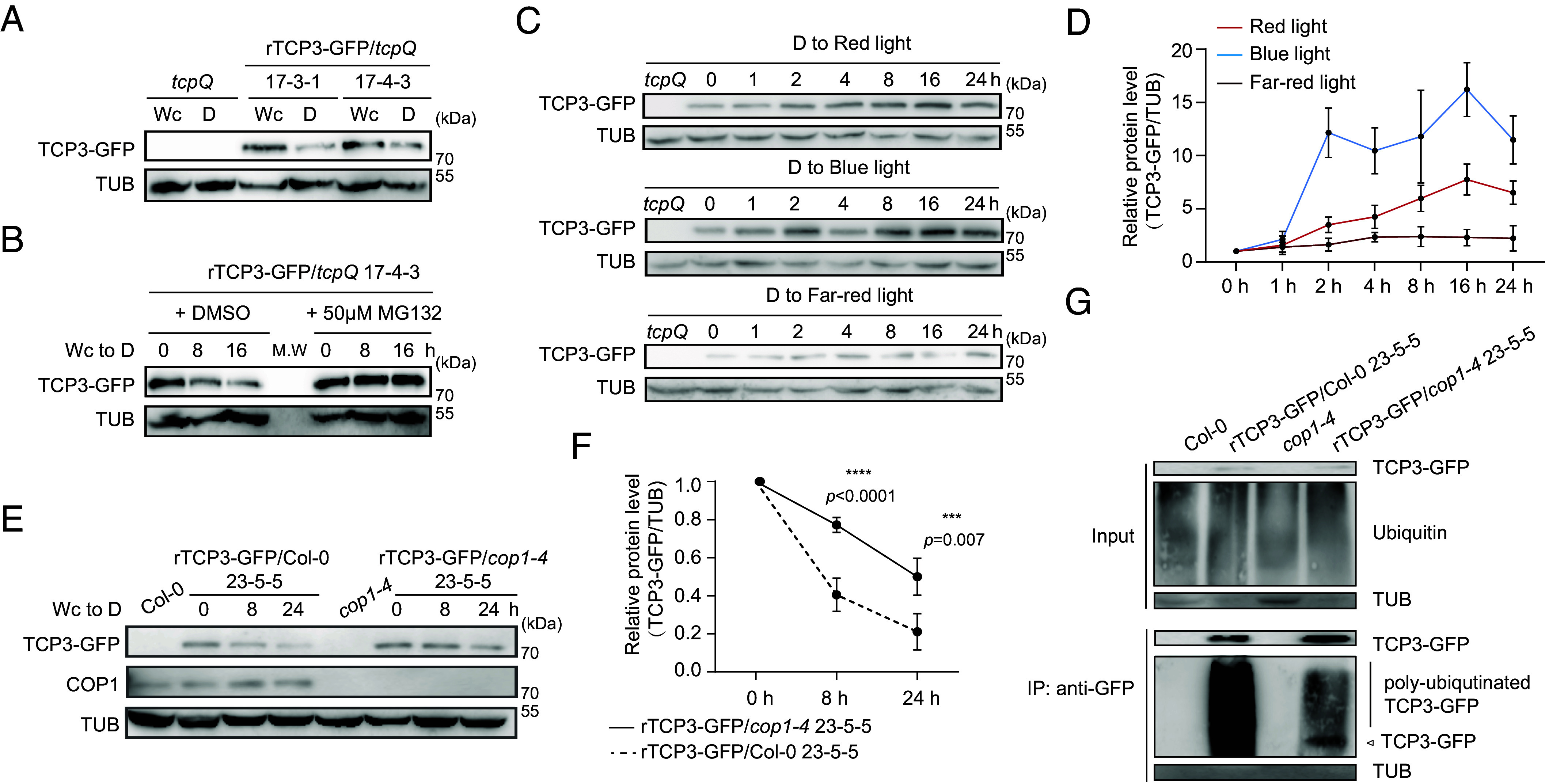
TCP3 is degraded in darkness in a COP1- and proteasome-dependent fashion (*A*) TCP3-GFP protein levels decrease in darkness. Homozygous rTCP3-GFP/*tcpQ* seedlings (lines 17-3-1 and 17-4-3) were grown under continuous white light (Wc) (100 μmol m^−2^ s^−1^) for 7 d and then transferred to darkness (D) for 12 h. TCP3-GFP and TUB proteins were detected using α-GFP and anti-α-tubulin antibodies, respectively. rTCP3 = miRNA319-resistant version of *TCP3*. (*B*) Degradation of TCP3-GFP depends on the 26S proteasome. Seedlings of rTCP3-GFP/*tcpQ* (line 17-4-3) were cultivated under Wc (100 μmol m^−2^ s^−1^) for 7 d, followed by treatment with 50 μM MG132 or DMSO and transfer to darkness for 8 or 16 h. (*C* and *D*) TCP3-GFP protein is stabilized by red, blue, and far-red light. Seedlings of rTCP3-GFP/*tcpQ* (line 17-4-3) grown in darkness for 7 d were exposed to red light (10 μmol m^−2^ s^−1^), blue light (10 μmol m^−2^ s^−1^), or far-red light (1 μmol m^−2^ s^−1^) for 1 to 24 h. TCP3-GFP and TUB proteins were detected (*C*) and quantified (*D*). TCP3-GFP/TUB levels in the 0 h samples were set to 1, respectively (*D*). Error bars represent SD of three biological replicates. (*E* and *F*) COP1 promotes the degradation of TCP3-GFP in darkness. The rTCP3-GFP/Col-0 transgene (from line 23-5-5) was crossed into the *cop1-4* mutant background (resulting in line 23-5-5/*cop1-4*). Homozygous 23-5-5 and 23-5-5/*cop1-4* were grown under Wc (100 μmol m^−2^ s^−1^) for 7 d and then transferred to darkness for 8 or 24 h. TCP3-GFP, COP1, and TUB levels were detected (*E*) and quantified (*F*). TCP3-GFP/TUB levels in the 0 h *COP1* wild type or *cop1-4* samples were set to 1, respectively (*F*). Error bars represent the SD from three independent experiments. Asterisks indicate significant differences between the wild-type and *cop1-4* backgrounds at the indicated time in darkness using Student’s *t* test. (*G*) In vivo ubiquitination of TCP3-GFP is enhanced by COP1. Homozygous rTCP3-GFP/Col-0 23-5-5, 23-5-5/*cop1-4*, and two controls (Col-0 and *cop1-4*) were grown for 7 d in Wc, then treated with 50 μM MG132 and transferred to darkness for 8 h. Proteins were pulled-down using α-GFP beads and detected with antibodies against GFP, ubiquitin, and α-tubulin.

To assess the impact of light conditions on TCP3 protein abundance, we used transgenic rTCP3-GFP/*tcpQ* seedlings to determine TCP3-GFP protein levels during light-to-dark transition. *Arabidopsis* seedlings were exposed to light for 7 d and then transferred to darkness for 12 h or kept under light for 12 h. Immunoblot analysis indicated that exposure to darkness decreased TCP3-GFP protein abundance (Fig. 3*A*). Treatment with MG132 effectively prevented the dark-induced reduction in TCP3-GFP levels (Fig. 3*B*), indicating that the TCP3 protein is degraded in darkness via the 26S proteasome pathway.

To investigate which wavelengths of light affect TCP3 protein abundance, we exposed dark-grown rTCP3-GFP/*tcpQ* seedlings to red light, blue light, or far-red light. Sampling occurred after 1, 2, 4, 8, 16, and 24 h of light exposure (Fig. 3*C*). The immunoblot results show that all three light conditions enhanced TCP3-GFP protein accumulation, with red and blue light being more effective than far-red light (Fig. 3 *C* and *D*).

### COP1 Promotes the Degradation of TCP3 After Light-to-Dark Transition.

Since TCP3 is degraded in darkness, we hypothesized that the E3 ubiquitin ligase COP1 may cause TCP3 degradation. To test this hypothesis, we first generated transgenic lines overexpressing TCP3-GFP in the Col-0 background and then selected two independent homozygous rTCP3-GFP/Col-0 lines (rTCP3-GFP/Col-0: lines 23-5-5 and 23-13-2). These lines were then crossed with the *cop1-4* mutant to obtain homozygous transgenic rTCP3-GFP/*cop1-4* lines (lines 23-5-5/*cop1-4* and 23-13-2/*cop1-4*). We did not use the previously generated rTCP3-GFP*/tcpQ* lines for these crosses because additional segregation of the four *tcp* mutations would have majorly complicated the generation of respective *cop1-4* lines in an otherwise uniform background.

We analyzed the TCP3-GFP protein abundance in the Col-0 and *cop1-4* backgrounds during light-to-dark transition. The degradation of TCP3-GFP in the *cop1-4* background was significantly slower than in the Col-0 background. Specifically, in the Col-0 background, the abundance of TCP3-GFP fell below 50% after 8 h of darkness, whereas it took approximately 24 h to reach this level in the *cop1-4* background (Fig. 3 *E* and *F*). In seedlings that were continuously grown in darkness or white light for 7 d, the *cop1-4* mutation also led to higher TCP3-GFP levels (*SI Appendix*, Fig. S6 *A* and *B*). Additionally, we compared the polyubiquitination levels of TCP3-GFP between Col-0 and *cop1-4* backgrounds. The transgenic seedlings were grown under light and then transferred to darkness in the presence of MG132. TCP3-GFP protein was immunoprecipitated using anti-GFP antibodies, and polyubiquitination levels were detected with an anti-ubiquitin antibody. The results show a much stronger polyubiquitination of TCP3-GFP in the Col-0 background when compared to the *cop1-4* background (Fig. 3*G*). Our findings therefore indicate that COP1 promotes the degradation of the TCP3 protein in darkness through polyubiquitination.

### TCP3 and COP1 Have Opposite Effects on Anthocyanin Accumulation.

To assess the biological relevance of COP1-induced TCP3 degradation, we investigated anthocyanin accumulation in *Arabidopsis* seedlings. Previous studies have demonstrated that TCP3 enhances anthocyanin accumulation, while COP1 reduces anthocyanin biosynthesis (34, 35, 44, 45). We first confirmed the opposing effects of TCP3 and COP1 on anthocyanin accumulation. We compared anthocyanin levels in Col-0, *tcpQ*, rTCP3-GFP/*tcpQ,* and *cop1-4* seedlings grown under continuous white light. The results indicate that, compared to Col-0, *tcpQ* seedlings exhibited reduced anthocyanin levels, whereas *cop1-4* seedlings showed increased anthocyanin levels. All three rTCP3-GFP/*tcpQ* lines showed higher anthocyanin levels than the *tcpQ* mutant progenitor, indicating that TCP3-GFP complemented the *tcpQ* mutant phenotype (*SI Appendix*, Fig. S7*A*). Transcript levels of anthocyanin biosynthesis structural genes, including *CHS*, *CHI*, *DFR,* and *LDOX*, were consistent with the observed anthocyanin levels (*SI Appendix*, Fig. S7 *B*–*F*). In contrast, the transcript levels of two transcription factors, *PAP1* and *PAP2*, which induce the expression of the structural genes, did not differ among the various genotypes (*SI Appendix*, Fig. S7 *G* and *H*). Taken together, these results show that TCP3 is a positive regulator of anthocyanin accumulation under the conditions used in these experiments.

### *TCP3* Genetically Interacts with *COP1* in the Accumulation of Anthocyanins.

**Fig. 4.**
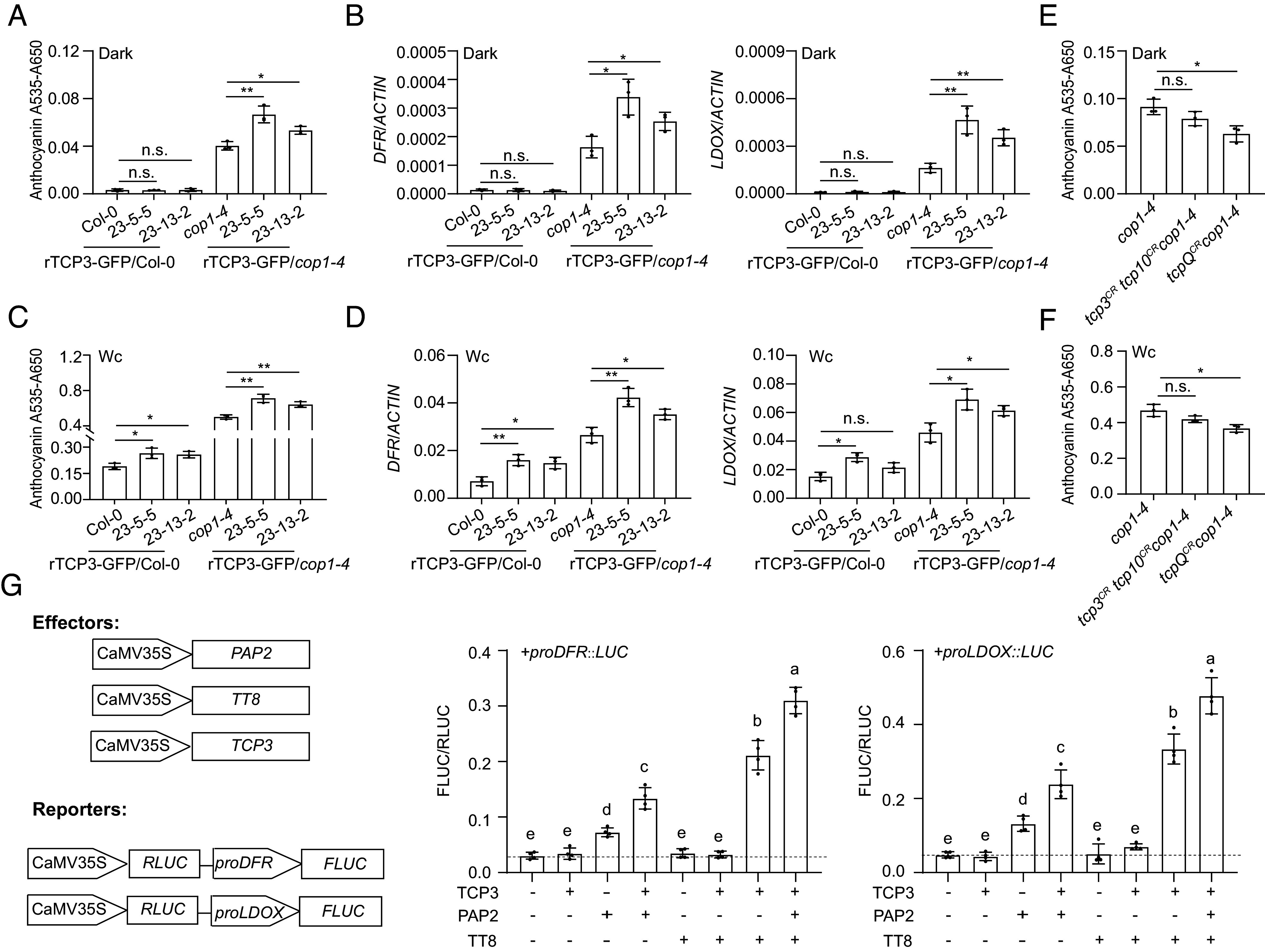
TCP3 promotes anthocyanin biosynthesis in the COP1 pathway. (*A*) Anthocyanin content of *Arabidopsis* seedlings of the indicated genotypes grown in darkness for 9 d. Error bars represent the SD from three biological replicates. Asterisks denote significant differences between the indicated pairs (Student’s *t* test, **P < 0.01, *P < 0.05, n.s. = not significant). (*B*) Transcript levels of *DFR*, *LDOX* in seedlings grown in darkness for 7 d. Transcript levels were normalized to *ACTIN*. Statistical analysis as in *A* (n = 3). (*C*) Anthocyanin content of *Arabidopsis* seedlings of the indicated genotypes grown in white light (Wc) for 9 d. Statistics as in *(A*). (*D*) Transcript levels of *DFR*, *LDOX* in seedlings grown in Wc for 7 d. Statistics as in (*A*). (*E* and *F*) Anthocyanin content of *Arabidopsis* seedlings of *cop1-4* and two *cop1-4 tcp* CRISPR-Cas9 mutants. *tcp3^CR^ tcp10^CR^* carries CRISPR mutations in *TCP3* and *TCP10; tcpQ^CR^* carries CRISPR mutations in *TCP2*, *TCP3*, *TCP4,* and *TCP10*. Anthocyanin content of seedlings grown in darkness (*E*) or Wc (*F*) for 9 d. Statistical analysis as in *A* (n = 3). (*G*) TCP3 enhances the effect of MBW proteins on *DFR* and *LDOX* promoter activities. Plasmid combinations expressing the indicated effector proteins were coinfiltrated with reporter plasmids into tobacco leaves, and FLUC and RLUC activities were determined on the third day postinfiltration. + indicates presence and − indicates absence of effector plasmids. Error bars represent the standard deviation from four biological replicates. Different letters denote significant differences between genotypes; genotypes sharing the same letter do not significantly differ, as determined by one-way ANOVA (*P* < 0.05).

Based on our finding that COP1 negatively regulates TCP3 protein stability, we predicted that the *cop1-4* mutation enhances the anthocyanin-promoting phenotype of TCP3 overexpression. To test this prediction, we compared the anthocyanin content of two TCP3-GFP lines in the Col-0 and *cop1-4* mutant backgrounds. When grown in darkness, neither Col-0 nor the two rTCP3-GFP/Col-0 lines accumulated detectable anthocyanins. In contrast, rTCP3-GFP/*cop1-4* lines accumulated more anthocyanin than *cop1-4* (Fig. 4*A*). This shows that TCP3 overexpression only enhanced anthocyanin accumulation in the *cop1-4* mutant background of dark-grown seedlings and not in a *COP1* wild-type background. Thus, *TCP3* genetically interacts with *cop1-4* which is consistent with our observation that the *cop1-4* mutation stabilizes the TCP3 protein in darkness (Fig. 3*E*). The transcript levels of anthocyanin biosynthesis structural genes (*CHS*, *CHI*, *DFR,* and *LDOX*) correlated with the respective anthocyanin levels while the abundance of *PAP1* and *PAP2* transcripts was extremely low and did not differ among all genotypes (Fig. 4*B* and *SI Appendix*, Fig. S8*A*). Also, the *cop1-4* mutation did not affect *rTCP3-GFP* transcript levels (*SI Appendix*, Fig. S8*A*).

When seedlings were grown in continuous light, both independent rTCP3-GFP/Col-0 lines accumulated higher anthocyanin levels compared to Col-0 wild type (Fig. 4*C*), which is in agreement with the results observed in rTCP3-GFP/*tcpQ* lines (*SI Appendix*, Fig. S7*A*). Also, both rTCP3-GFP/*cop1-4* lines accumulated almost 1.5-fold more anthocyanin than *cop1-4* (Fig. 4*C*). Transcript levels of *CHS*, *CHI*, *DFR,* and *LDOX* mirrored the anthocyanin content; these transcripts were upregulated following the introduction of *cop1-4*. Again, the *PAP1* and *PAP2* transcript levels were not upregulated by *rTCP3-GFP* overexpression nor by the *cop1-4* mutation (Fig. 4*D* and *SI Appendix*, Fig. S8*B*). It is worth mentioning that rTCP3-GFP/*cop1-4* inflorescences were purple at the stem, pedicel, and sepal tissues. This phenotype was barely observed in rTCP3-GFP/Col-0 and not observed in Col-0 and *cop1-4* (*SI Appendix*, Fig. S9).

To further investigate whether TCP3 acts in the COP1 pathway, we generated *tcp cop1-4* mutants. This was not possible by crossing *tcpQ* with *cop1-4* because *TCP10* and *COP1* loci are very closely linked. We therefore used CRISPR-Cas9 to mutate *TCP*s in the *cop1-4* background. Given the potential functional redundancy among TCPs, we selected triple and quintuple mutants, including *tcp3*, to better understand their roles. Through genotyping, we obtained two lines: *tcp3^CR^ tcp10^CR^cop1-4* (mutated *TCP3* and *TCP10*) and *tcpQ^CR^cop1-4* (mutated *TCP2*, *TCP3*, *TCP4,* and *TCP10*) (*SI Appendix*, Fig. S10). We quantified anthocyanin content in these lines. The results show that *tcpQ^CR^* mutations significantly reduced the anthocyanin content in *cop1-4* under both dark and light conditions, while in the presence of only two *tcp* mutations, the reduction in anthocyanin content when compared to *cop1-4* was not statistically significant (Fig. 4 *E* and *F*).

Taken together, our findings indicate that *cop1-4* enhances anthocyanin biosynthesis through TCPs, a phenotype that is particularly evident under dark conditions. Thus, COP1 inhibits anthocyanin accumulation at least in part through inhibiting the actions of these TCPs.

### TCP3 Enhances the Effect of MBW Proteins.

To further understand how TCP3 increases anthocyanin accumulation, we conducted dual-luciferase assays in transfected tobacco leaves (46). Firefly luciferase was expressed under the control of *DFR* or *LDOX* promoters in the presence or absence of coexpressed TCP3 and members of the MBW complex. This allowed us to investigate whether TCP3 coacts with the MBW complex, as suggested previously (34). The results indicate that the MYB transcription factor PAP2 alone enhanced the promoter activities of *DFR* and *LDOX*, while rTCP3 and the bHLH TT8 alone had no effect on *proDFR::LUC* and *proLDOX::LUC* expression. The coexpression of rTCP3 significantly increased the activities of PAP2 and TT8, resulting in a marked induction of *DFR* and *LDOX* promoters (Fig. 4*G*). This suggests that TCP3 might enhance the activity of the MYB–TT8 complex.

### TCP3 Plays a More Prominent Role in Promoting Flowering in LD Compared to SD.

Previous research has demonstrated that TCP2, TCP3, TCP4, and TCP10 are involved in photoperiodic flowering, acting as flowering-promoting factors, while the COP1/SPA complex functions as a negative regulator in this process (31, 32, 47–50). To investigate the activity and stability of the TCP3 protein during flowering, we grew three rTCP3-GFP/*tcpQ* lines and respective controls in LD and SD. Under LD conditions, we observed that both *tcp3/4* and *tcpQ* mutants exhibited a late-flowering phenotype compared to Col-0, with more rosette leaves formed at the time of bolting and an increased number of days needed to bolting, as reported previously (*SI Appendix*, Fig. S11 *A* and *B*) (31, 32). In contrast, the *cop1-4* mutant exhibited the previously reported early-flowering phenotype (*SI Appendix*, Fig. S11 *A* and *B*) (49, 50). The expression of *rTCP3-GFP* in transgenic *tcpQ* accelerated flowering, indicating partial complementation of the *tcpQ* mutant phenotype (*SI Appendix*, Fig. S11 *A* and *B*). The curled leaf phenotype of *tcpQ* mutants was also partially complemented by *rTCP3-GFP* (*SI Appendix*, Fig. S11*C*).

Under SD conditions, the *tcp3/4* and *tcpQ* mutants exhibited a weaker phenotype than under LD conditions (*SI Appendix*, Fig. S11 *D* and *E*). *tcp3/4* mutants flowered with the same number of leaves as Col and the *tcpQ* mutant showed only a slight delay in flowering time when analyzing the number of rosette leaves formed at bolting. Nevertheless, *tcp3/4* and *tcpQ* mutants flowered later than the wild type with respect to DAG (*SI Appendix*, Fig. S11 *D* and *E*). This indicates that these *tcp* mutants grew more slowly than the wild type, while flowering at a similar stage of development. The expression of *rTCP3-GFP* in *tcpQ* had little effect in rescuing the curled leaf phenotype of *tcpQ* under SD, and statistical analysis of flowering time showed no significant difference between the rTCP3-GFP/*tcpQ* lines and *tcpQ* (*SI Appendix*, Fig. S11 *D*–*F*). This demonstrates that in the *tcpQ* background, expression of *rTCP3-GFP* accelerated *Arabidopsis* flowering under LD but had less function under SD.

To elucidate the underlying mechanism of TCP3 action, we determined the mRNA levels of *CONSTANS* and *FLOWERING LOCUS T* (*FT*). In LD, the *CO* and *FT* transcript levels were lower in *tcpQ* than in Col wild type which is consistent with the late-flowering phenotype of *tcpQ*. In rTCP3-GFP/*tcpQ* lines, *CO* and *FT* mRNA levels were higher than in *tcpQ*, indicating that *TCP3* led to a higher expression of *CO* and *FT* in LD (*SI Appendix*, Fig. S11*G*). In SD, the *CO* transcript levels were lower in the *tcpQ* mutant than in the wild type, while *FT* transcript levels were unchanged (*SI Appendix*, Fig. S11*H*). However, *rTCP3-GFP* overexpression in the *tcpQ* mutant background did not lead to an increase in *CO* or *FT* transcript levels (*SI Appendix*, Fig. S11*H*) which is consistent with the failure of *rTCP3-GFP* to rescue the slight early flowering of *tcpQ*. Thus, the slight early flowering of *tcpQ* in SD is likely due to the lack of *TCP2*, *TCP4,* and/or *TCP10*, and less due to the lack of *TCP3*. Taken together, the results show that TCP3 is a positive regulator of flowering time in LD, but less in SD, and likely acts via increasing *CO* and *FT* expression in LD.

### LD Causes an Increase in TCP3 Protein Levels.

We next investigated whether the LD-enhanced effect of *rTCP3-GFP* may be due to a higher transcript accumulation of *rTCP3-GFP* and/or a higher protein accumulation of TCP3-GFP in LD than in SD. *SI Appendix*, Fig. S11 shows that *TCP3-GFP* transcript levels were approx. 30% higher in LD than in SD, suggesting that daylength has a mild effect on the transcript stability of *rTCP3-GFP* derived from the *35S::rTCP3-GFP* transgene. We also isolated proteins at corresponding time points from aliquots of the identical plant material that we used for the isolation of RNA. TCP3-GFP protein was clearly detected in LD-grown plants of all three transgenic lines, with protein levels increasing under light conditions and decreasing after transition to darkness (*SI Appendix*, Fig. S11*I*). In SD-grown plants, the TCP3-GFP protein was not detectable in line 17-2-6, and only weak TCP3-GFP signals were observed at ZT7 and ZT15 in lines 17-3-1 and 17-4-3 (*SI Appendix*, Fig. S11*J*). To determine whether the reduced *rTCP3-GFP* mRNA levels in SD contributed to the lower TCP3-GFP protein accumulation in SD compared to LD, we included the ZT7 protein extract from LD-grown plants (undiluted and 10-fold diluted) as a control. The results show that the TCP3-GFP protein levels in SD-grown plants were less than 10% of that from LD-grown plants, indicating that not only mRNA levels but mainly TCP3 protein levels were negatively regulated by SD conditions when compared to LD conditions (*SI Appendix*, Fig. S11*J*). In conclusion, TCP3 protein accumulated under LD conditions to promote flowering by increasing the expression of *CO* and *FT*. Under SD conditions, the protein levels of TCP3 were very low, preventing TCP3-GFP from restoring the *tcpQ* phenotype and hindering TCP3’s ability to promote flowering.

### The *cop1-4* Mutation Enhances the Effect of TCP3 on Flowering Time and Increases TCP3 Protein Levels in Leaves.

**Fig. 5.**
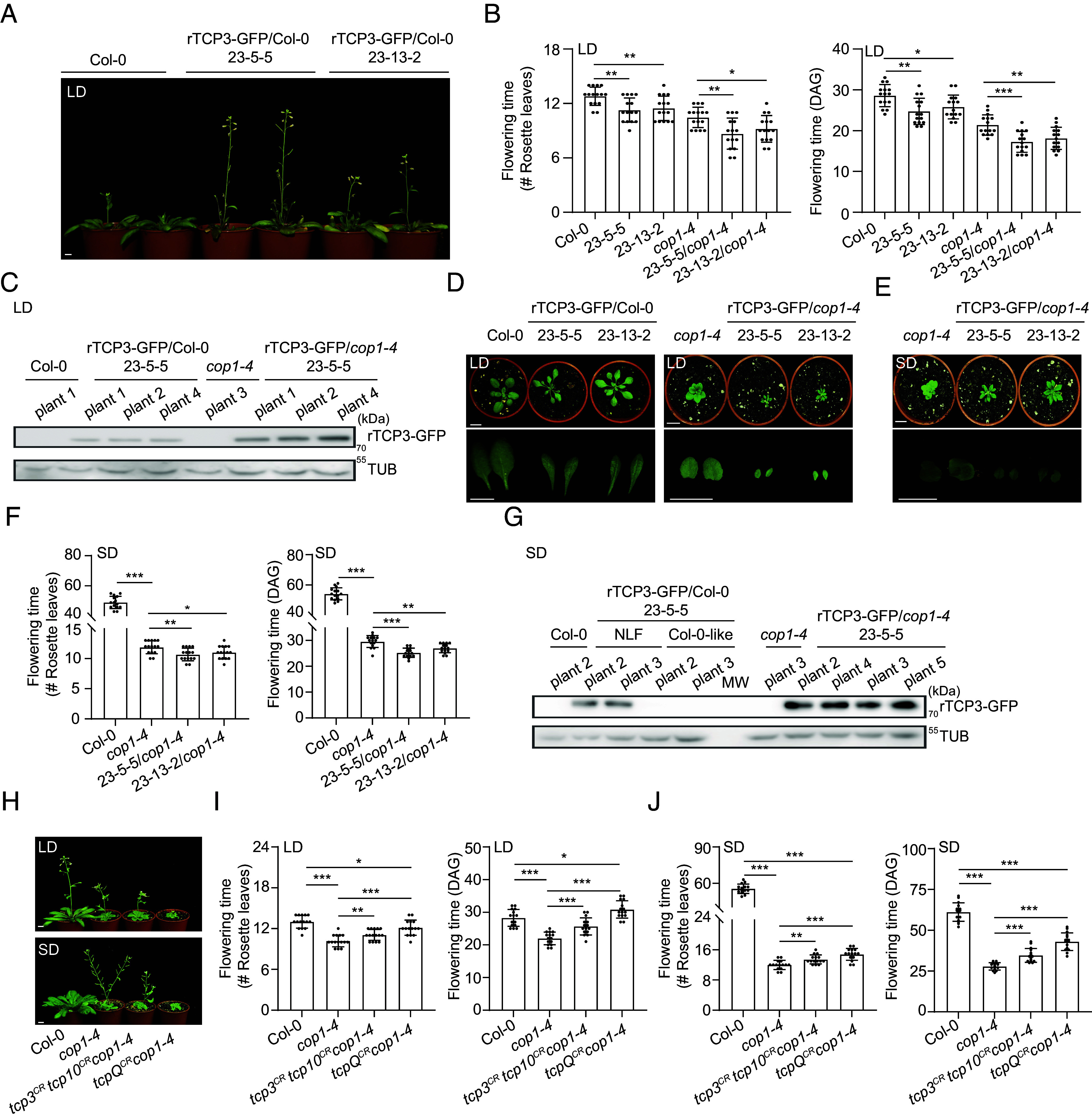
COP1 negatively regulates TCP3 protein abundance during flowering. (*A* and *B*) *cop1-4* enhances the effect of TCP3 on flowering time in LD. (*A*) Visual phenotypes of Col-0 and two rTCP3-GFP/Col-0 lines cultivated in LD for 4 wk. (*B*) Rosette leaf numbers and days after germination (DAG) at bolting time of rTCP3-GFP lines in Col-0 wild-type and *cop1-4* mutant backgrounds. The respective *rTCP3-GFP* transgenes were transferred from the Col-0 to the *cop1-4* background by crossing. Error bars represent the SD of 15 plants. Asterisks denote significant differences between the indicated pairs (Student’s *t* test, ***P < 0.001, **P < 0.01, *P < 0.05). (*C*) TCP3 protein levels in Col-0- and *cop1-4* backgrounds in LD. TCP3-GFP protein levels were analyzed in extracts from individual plants of the indicated genotypes grown in LD and harvested at ZT15. Individual Col-0 and *cop1-4* plants were used as negative controls. TCP3-GFP and TUB proteins were detected using α-GFP and anti-α-tubulin antibodies, respectively. *TCP3* transcript levels using tissue aliquots of the same individual plants are shown in *SI Appendix*, Fig. S12*B*, demonstrating that *cop1-4* does not affect *TCP3* transcript levels. (*D*) Visual phenotypes of rTCP3-GFP/Col-0 and rTCP3-GFP/*cop1-4* grown in LD. Plants in the Col background were 2 wk old, in the *cop1-4* background 3 wk old due to slower growth. (Scale bar, 1 cm.) (*E* and *F*) TCP3 accelerates *cop1-4* flowering time in SD. (*E*) Visual phenotype of *cop1-4* and rTCP3-GFP/*cop1-4* (Lines 23-5-5/*cop1-4* and 23-13-2/*cop1-4*) grown in SD for 4 wk. (Scale bar, 1 cm.) (*F*) Rosette leaf numbers and DAG at the time of bolting of the indicated genotypes grown in SD. Statistical analysis as in (*B*), n = 15 plants. (*G*) TCP3 protein levels in Col-0 and *cop1-4* backgrounds grown in SD. TCP3-GFP protein levels were analyzed in extracts from individual plants of the indicated genotypes grown in SD and harvested at ZT7. Individual Col-0 and *cop1-4* plants were used as negative controls. *TCP3* transcript levels using tissue aliquots of the same individual plants are shown in *SI Appendix*, Figs. S13*B* and S14*F*. NLF: plants showing the narrow-leaf phenotype reminiscent of TCP3 overexpression. Col-0: plants exhibiting a wild-type leaf phenotype. (*H*–*J*) *tcp* mutations delay flowering in *cop1-4*. (*H*) Visual phenotypes of Col-0 wild type, *cop1-4,* and two *tcp cop1-4* CRISPR-Cas9 mutants grown in LD (*Top*) and SD (*Bottom*). *tcp3^CR^ tcp10^CR^* carries CRISPR mutations in *TCP3* and *TCP10*; *tcpQ^CR^* carries CRISPR mutations in *TCP2*, *TCP3*, *TCP4,* and *TCP10*. Plants grown in LD were 4 wk old, while plants in SD were 6 wk old. (Scale bar, 1 cm.) (*I* and *J*). Flowering time was quantified as rosette leaf numbers and DAG at bolting time in LD (*I*) and SD (*J*). Statistical analysis as in (*B*) (n = 15).

The above results suggest that SD may downregulate TCP3 protein accumulation when compared to LD. We therefore investigated the role of the ubiquitin ligase COP1 in regulating TCP3 protein levels and TCP3 activity as an inducer of flowering in LD. To this end, we analyzed TCP3-GFP protein levels and flowering time in rTCP3-GFP/Col-0 (lines 23-5-5 and 23-13-2) and in rTCP3-GFP/*cop1-4* lines in which the *rTCP3-GFP* transgene had been crossed from the Col wild-type background into the *cop1-4* mutant background (23-5-5/*cop1-4* and 23-13-2/*cop1-4*). rTCP3-GFP/Col-0 plants flowered earlier than Col-0 under LD conditions, confirming that TCP3 promotes flowering in LD (Fig. 5 *A* and *B*). rTCP3-GFP/*cop1-4* plants flowered earlier than the *cop1-4* mutant, suggesting that the *cop1-4* mutation enhances the effect of TCP3-GFP overexpression on flowering time (Fig. 5 *B* and *D*). Indeed, the early flowering correlated with higher rTCP3-GFP protein levels in the *cop1-4* mutant background when compared to the Col background, using protein isolated from individual plants as biological replicates (Fig. 5*C*). Similarly, *CO* transcript levels were much higher in RNA isolated from the same individual plants of LD-grown rTCP3-GFP*/cop1-4* than in rTCP3-GFP/Col lines (*SI Appendix*, Fig. S12*A*). Thus, TCP3-GFP protein levels were positively correlated with *CO* transcript levels and flowering time. In contrast, the *cop1-4* mutation did not change *rTCP3-GFP* transcript levels (*SI Appendix*, Fig. S12*B*). This suggests that the *cop1-4* mutation leads to reduced degradation of the TCP3-GFP protein in LD, thereby enhancing *CO* expression which causes earlier flowering. *rTCP3-GFP* overexpression also reduced the plant size and changed the leaf shape to more narrow leaves in LD. This was observed in both the Col and the *cop1-4* mutant backgrounds (Fig. 5*D*).

When grown in SD conditions, two independent rTCP3-GFP/*cop1-4* lines flowered earlier than *cop1-4*, indicating that in a *cop1-4* mutant background *rTCP3-GFP* overexpression further accelerates flowering also in SD (Fig. 5 *E* and *F*). This earlier flowering correlated with increased *CO* transcript levels in rTCP3-GFP/*cop1-4* plants compared to *cop1-4* mutant plants (*SI Appendix*, Fig. S13*A*). TCP3-GFP protein levels were also much higher in the *cop1-4* mutant background than in the Col wild-type background (Fig. 5*G*). However, we noticed that both *rTCP3-GFP* overexpression lines in the Col wild-type background consistently displayed two different phenotypes in SD: some plants showed no change in leaf shape or plant size (“Col-like”), flowered at the same time as the wild type, showed no change in *CO* transcript levels when compared to the wild type and almost no detectable accumulation of TCP3-GFP protein (*SI Appendix*, Fig. S14 *A*–*D* and Fig. 5*G*), as we had observed when analyzing TCP3-GFP lines in the *tcpQ* mutant background in SD (*SI Appendix*, Fig. S11 *D*–*F* and *J*). In contrast, some rTCP3-GFP/Col plants did show an effect of TCP3-GFP overexpression in SD. These plants had narrow leaves (“NLF”), flowered earlier, and had higher *CO* transcript levels than the wild type (*SI Appendix*, Fig. S14 *A*–*E*). They also accumulated some TCP3-GFP protein in SD, though still considerably less than rTCP3-GFP/*cop1-4* lines (Fig. 5*G*). When analyzing individual plants, we found that plants lacking a TCP3-GFP overexpression phenotype showed lower *rTCP3-GFP* transcript levels than those showing a TCP3-GFP overexpression phenotype in SD (*SI Appendix*, Figs. S13*B* and S14*F*). This suggests that high *rTCP3-GFP* expression followed by TCP3-GFP accumulation is needed to cause the narrow leaf phenotype and early flowering in SD. We do not know the reason for this plant-to-plant variability in rTCP3-GFP expression and why we only observed this in SD and not in LD (*SI Appendix*, Fig. S15 *A* and *B*). However, it is evident that TCP3-GFP protein only accumulates in SD when TCP3-GFP expression is very high, thereby likely—at least in part—escaping COP1-mediated degradation.

To investigate whether *cop1-4* promotes flowering through TCPs, we examined flowering time of *cop1-4* and two previously mentioned *cop1-4 tcp* CRISPR-Cas9 lines (*tcp3^CR^ tcp10^CR^cop1-4* and *tcpQ^CR^cop1-4*) in LD and SD (*SI Appendix*, Fig. S10). We found that as the number of mutated *TCP* loci increased, flowering in *cop1-4* plants was progressively delayed. Notably, mutating just two *TCP*s was sufficient to delay flowering in *cop1-4*, and this delay was observed under both LD and SD (Fig. 5 *H*–*J*). These findings indicate that TCPs have additive effects in promoting flowering. In summary, TCPs play a functional role in the COP1 pathway.

Taken together, our results demonstrate that *cop1-4* promotes flowering in part through inhibiting TCPs. Specifically, the *cop1-4* mutation causes a stabilization of the TCP3 protein, thereby causing an increased expression of *CO* and earlier flowering in both LD and SD.

### The Action of TCP3 on Flowering Time Depends on *CO*.

Our data suggest that TCP3 accelerates flowering by increasing the expression of the *CO* gene. To corroborate this hypothesis, we conducted dual luciferase assays in transfected tobacco leaves (*SI Appendix*, Fig. S16*A*). Coexpression of *TCP3* increased the activity of the *proCO::LUC* construct, indicating that TCP3 has a promoting effect on the *CO* promoter. To investigate whether TCP3 action depends on *CO* we constructed TCP3-GFP/*co-10* plants by crossing the *TCP3-GFP* transgene of two independent *rTCP3-GFP* lines into the *co-10* mutant background (23-5-5/*co-10* and 23-13-2/*co-10*). TCP3-GFP was unable to accelerate flowering in the *co-10* mutant background (*SI Appendix*, Fig. S16 *B* and *C*). These experiments demonstrate that TCP3-dependent expression of *CO* promotes flowering in LD.

## Discussion

**Fig. 6.**
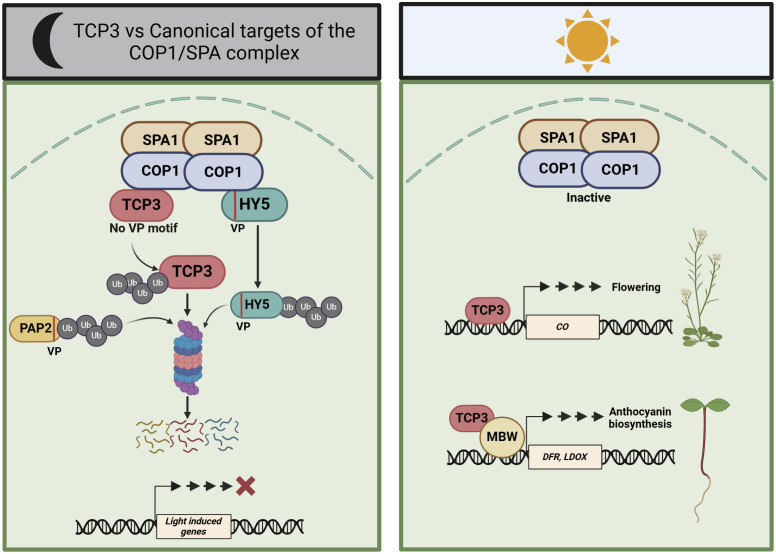
A working model for the COP1/SPA-TCP3 pathway. In darkness (*Left* panel), the COP1/SPA complex promotes the ubiquitination and subsequent degradation of HY5 and PAP2 (as examples for canonical, VP-containing substrates) and TCP3 (as a noncanonical substrate lacking a VP motif). Their degradation prevents photomorphogenesis in darkness. In the light (*Right* panel), the activity of the COP1/SPA complex is inhibited. This inhibition prevents the ubiquitination and degradation of TCP3, allowing TCP3 to accumulate and to promote flowering and anthocyanin biosynthesis by inducing the expression of *CONSTANS* (*CO*) and anthocyanin biosynthesis genes, respectively.

TCP transcription factors represent a family of proteins with very diverse functions in plant growth and development (29). Here, we have shown that the class II TCP transcription factor TCP3 interacts with the COP1/SPA ubiquitin ligase in vivo, thereby causing degradation of TCP3 in darkness and preferentially under short day conditions. Importantly, TCP3 lacks a canonical VP motif, which is a previously identified COP1-interacting motif present in many COP1/SPA substrates. This indicates that COP1/SPA also recognizes motifs other than VP. We further defined the activity of COP1 in TCP3-mediated control of anthocyanin accumulation and flowering time. Taken together, our results have uncovered TCP as a transcription factor family that is targeted by COP1/SPA via a noncanonical protein–protein interaction (Fig. 6).

### TCP3 Lacking a Canonical VP Motif Is a Substrate of the COP1/SPA1 Ubiquitin Ligase.

We have shown through Y2H, LCI, FRET-FLIM, in vivo coimmunoprecipitation, and in vitro pull-down that TCP3 directly interacts with COP1 and SPA1 proteins (Fig. 1 *A*–*E*). We have further shown that TCP3 levels decrease in darkness in a COP1- and proteasome-dependent fashion, while TCP3 is stabilized by exposure to red, far-red, and blue light (Fig. 3 *A*–*F*). Similarly, polyubiquitination of TCP3 in *Arabidopsis* seedlings is strongly dependent on COP1 (Fig. 3*G*). Taken together these results support that TCP3 is a degradation substrate of the COP1/SPA complex.

COP1 is an evolutionarily conserved ubiquitin ligase, with its three structural domains present across mammals and plants (9, 15). Its WD-repeat domain interacts with substrate transcription factors as well as with photoreceptors, such as CRY1, CRY2, and UVR8 which inhibit COP1 activity in the light by displacing substrates from COP1 (6, 16, 17). These photoreceptors and substrates contain a common VP motif responsible for direct interaction with the WD-repeat domain of COP1 (6, 14, 16). Further crystallographic studies by Lau et al. (6) and Uljon et al. (15) revealed that the core residues of the VP motif form the center of the COP1 binding site in *Arabidopsis* and human interactors, with the VP motif residues linking these proteins to COP1 by deeply inserting into the VP binding pocket of COP1. Our study identified TCP3 as a COP1 substrate that lacks a canonical VP motif (Figs. 1 *A*–*E* and 2 *A*–*F* and *SI Appendix*, Fig. S2*C*). Similar to TCP3, both TCP4 and TCP10 interact with COP1/SPA1 in the yeast two-hybrid system (*SI Appendix*, Fig. S4*E*). Notably, TCP4, like TCP3, lacks the VP motif, while the interaction between TCP10 and COP1 remains unaffected even when the putative VP motif is mutated (Fig. 2 *A* and *B* and *SI Appendix*, Fig. S2*C*). Similar to previously discovered substrates of COP1, TCP3 interacts with the WD-repeat domains of COP1 and SPA1 (*SI Appendix*, Fig. S4 *B*–*D*). Surprisingly, when comparing the COP1 WD-repeat domain regions interacting with TCP3 and VP-containing substrates, we found that TCP3 appears to share the same binding region as PAP2, a VP-containing substrate (6, 14, 44) (Fig. 2 *E* and *F*). This suggests that even in the absence of a VP domain, TCP proteins may interact with COP1 in a manner similar to VP-containing substrates. This finding is of importance because it suggests that UVR8 and CRY2 photoreceptors may stabilize also TCPs by competitively displacing them from the COP1 WD-repeat domain (6, 16). Notably, TCP3 is not the only COP1 substrate that lacks a VP domain; other examples include BIT1, EBF2, and TZP (51–53). It will be particularly intriguing to explore these interactions in greater depth through protein crystallization and structural analyses. Another observation is that the N-terminal domain of SPA1 containing the kinase domain also interacted with TCP3 (*SI Appendix*, Fig. S4*D*). This raises the question whether SPA1 not only participates in the degradation of TCP3 but also phosphorylates TCP3. Indeed, the close TCP3 homolog TCP4 was previously shown to be potentially phosphorylated at multiple sites in vivo (31).

We experimentally defined that the TCP domain of TCP3 mediates the interaction with COP1 and SPA1 (*SI Appendix*, Fig. S5 *A*–*D*). The TCP domain of TCP transcription factors is generally associated with dimerization and DNA binding of TCP proteins (22). This domain is conserved among TCP transcription factors, implying that other TCPs, besides TCP3, may also interact with the COP1/SPA complex and, therefore, present degradation substrates of COP1/SPA. Indeed, we found that TCP4 and TCP10 - two TCPs very closely related to TCP3 - also interact with COP1 and SPA1 in the yeast two hybrid assay. Previous reports showed that TCP2 and TCP4 are degraded in darkness and stabilized by light (38, 47). Also, TCP5, TCP13, and TCP17 were reported to be stabilized by light (54), though another report showed accumulation of TCP17 in the dark and degradation in the light (55). With the exception of TCP17, these TCPs have in common that they belong to the CIN-type class II TCPs. It remains to be clarified whether the class I TCP17 is indeed stabilized or destabilized by light conditions (54, 55). Thus, it remains to be investigated whether there is a difference in light regulation between class I and class II TCPs. Anyhow, it is apparent that the TCP domain has multiple functions: dimerization, DNA-binding, and being a degron for COP1/SPA. Moreover, it is involved in other protein interactions. One example is the interaction between TCP5 and PIF4. Researchers found in yeast that the interaction between TCP5 and PIF4 was greatly weakened when the TCP domain was removed (56).

### TCP3 Is Involved in COP1/SPA1-Mediated Anthocyanin Biosynthesis.

The regulation of anthocyanins biosynthesis in *Arabidopsis* mainly depends on the MBW complex formed by the transcription factors MYB (PAP1, PAP2, MYB113 and MYB114), bHLH (GL3/EGL3/TT8), and the WD-repeat protein TTG1 (57). The MBW complex binds to the promoters of anthocyanin biosynthesis structural genes to promote their expression in light-grown plants (58). In dark-grown *Arabidopsis*, anthocyanin accumulation is suppressed by the COP1/SPA complex (11, 44, 45). TCP family members are also involved in anthocyanin accumulation in opposite ways: on the one hand, several CIN-TCPs are positive regulators of anthocyanin biosynthesis (34–36, 48). On the other hand, TCPs can also act as inhibitors of anthocyanin accumulation under excessive light conditions, likely mediated by redox regulation of TCP (37, 54). In dark-grown seedlings, TCP3 overexpression did not increase anthocyanin accumulation, unless we introduced the *cop1-4* mutation (Fig. 4*A*). This is consistent with the increased accumulation of TCP3 protein observed in the *cop1-4* background (*SI Appendix*, Fig. S6). In the light, the effect of TCP3 overexpression on anthocyanin levels was enhanced by the *cop1-4* mutation, indicating that COP1 reduces TCP3 function in the light (Fig. 4*C*).

In the *cop1-4* background, mutating TCPs led to a greater reduction in anthocyanin levels under both dark and light conditions as the number of mutated *TCP*s increased (Fig. 4 *E* and *F*). This indicates that TCPs play a promotive role in anthocyanin biosynthesis within the COP1 pathway. Thus, degradation of at least three distinct transcription factor families (HY5/HYH, PAP1/PAP2, and TCP3) are under the control of COP1/SPA to limit anthocyanin accumulation (9, 44).

It is worth noting that while anthocyanin content decreases with the reduction in the number of TCPs, the decrease is limited compared to the almost complete loss of anthocyanins observed when all four MYBs (*PAP1*, *PAP2*, *MYB113*, *MYB114*) are silenced in the *cop1-4* background (44). This could be explained in two ways. First, the limited reduction might be due to the insufficient number of mutated *TCPs*, and mutating additional *TCP*s could reveal their greater importance in anthocyanin biosynthesis. Second, it is possible that TCPs, while involved in anthocyanin biosynthesis, function as “assistants” rather than “primary players”. This is supported by our dual-luciferase assay results, which show that TCP3 alone cannot activate the expression of *DFR* or *LDOX* but requires the PAP2–TT8 complex (Fig. 4*G*). This is consistent with previous studies, showing that TCP3 can enhance the interaction between PAP2 and TT8 in yeast (34). This finding is moreover consistent with research in apple (*Malus domestica*), where it was demonstrated that MdTCP46 depends on MdMYB1 (homolog to PAP1) to activate the expression of *MdDFR* or *MdUF3GT* (59). Furthermore, the role of TCPs in either activating or repressing anthocyanin biosynthesis may rely on specific MYB proteins they interact with in a particular spatial or temporal context. Previous studies (34) have shown that TCP3 can also interact with the repressive protein MYBL2, suggesting that investigating the functional outcomes of different MYB-TCP combinations presents intriguing research.

### LD Regulates TCP3 Protein Accumulation Through COP1/SPA in the Control of Flowering Time.

Multiple members of the TCP family are involved in flowering time regulation. TCP2, 3, 4, and 10 of the class II TCP family promote flowering by inducing the expression of *CO*, while TCP5, 13, and 17 promote flowering by inducing the expression of *AP1* (31, 32, 60, 61). In the class I TCP family, TCP7, TCP14, and TCP15 promote flowering by inducing *SOC1* expression, while TCP18, TCP20, TCP22, and TCP23 are inhibitory factors of flowering time (61, 62). In agreement with prior research findings, we found that TCP3 is a positive regulator of flowering time and leads to higher transcript levels of *CO* (31, 32) (*SI Appendix*, Fig. S11 *A*, *B*, *D*, *E*, and *G*). The effect of TCP3-GFP overexpression on flowering time was dependent on the *CO* gene. Moreover, TCP3 coexpression activated the *CO* promoter in transient transactivation assays (*SI Appendix*, Fig. S16*A*). This is in agreement with a previous study showing that TCP3 can bind the *CO* promoter (31).

In our study, *tcp3/4* and *tcpQ* exhibited late flowering in LD, whereas only *tcpQ* showed late flowering in SD (*SI Appendix*, Fig. S11 *A*, *B*, *D*, and *E*). Overexpression of TCP3 in the *tcpQ* background can partially rescue the flowering time phenotype in LD but fails to do so in SD (*SI Appendix*, Fig. S11 *A*, *B*, *D*, and *E*). Here, we have shown that the preferential activity of TCP3 in LD correlates with TCP3 protein accumulation in LD, while TCP3 is mostly degraded under SD (*SI Appendix*, Fig. S11 *I* and *J*). In LD, TCP3 protein abundance is high during the light phase, while it declines upon transition to darkness, suggesting that light exposure stabilizes the TCP3 protein, as we had observed for seedlings (Fig. 3 *A* and *B* and *SI Appendix*, Fig. S11*I*). Degradation of TCP3 is reduced in leaves of the *cop1-4* mutant, indicating that the ubiquitin ligase COP1 targets TCP3 also during flowering process (Fig. 5*C*). In SD, COP1 also negatively regulates the protein stability of CO to inhibit flowering (50, 63, 64). Thus, our study shows that COP1 also participates in negatively regulating the stability of TCP3—in addition to CO—to allow earlier flowering in LD than in SD. The *tcp3/4* and *tcpQ* mutants also carry a mutation in the *TCP4* gene. TCP4 protein levels were previously shown not to be altered throughout the LD photoperiod, i.e. no decline in TCP4 protein levels was observed during the night in LD (31). However, TCP4 protein abundance in SD has not yet been analyzed. Interestingly, TCP3-overexpression also at least partially rescued the leaf shape phenotype only in LD and not in SD (*SI Appendix*, Figs. S11 *C* and *F*, S14*B*, and S15 *A* and *B*). This suggests that the light duration may also play a role in leaf shape determination.

## Materials and Methods

*tcp3/4*, *tcpQ*, *cop1-4,* and *co-10* (also called *co-SAIL*) were described previously (24, 43, 45, 64). Seedlings were grown on Murashige and Skoog (MS) media (without sucrose) in growth chambers equipped with light-emitting diode (LED) light sources (CLF Plant Climatics, Wertingen, Germany) producing white, red, far-red, or blue light at a temperature of 21 °C. For analysis of adult phenotypes, plants were grown on soil in a climate-controlled walk-in growth chamber under long day (16 h light/8 h darkness) or short day (8 h light/16 h darkness) at 21 °C. White light was supplied by Fluora L58W/77 fluorescent tubes. Fluence rates for each experiment are detailed in the corresponding figure legends.

To generate rTCP3-GFP overexpressing plants, the rTCP3-pFAST-R05 plasmid was introduced into *Agrobacterium tumefaciens* GV3101(PMP90), followed by Agrobacterium-mediated transformation of *tcpQ* or Col-0 plants via floral dip method. For the selection of transgenic lines, we utilized the RFP fluorescence in the pFAST plasmid as marker (65), with seed fluorescence detected using a ZEISS Axio Zoom fluorescence microscope (Carl Zeiss AG, Germany). For constructing of rTCP3-GFP/*cop1-4* plant, pollen from mature rTCP3-GFP/Col-0 (23-5-5 and 23-13-2) flowers was placed onto the stigma of the emasculated *cop1-4* plants (23-5-5/*cop1-4* and 23-13-2/*cop1-4*). The RFP fluorescence and dwarf architecture were used to select *rTCP3-GFP/cop1-4* plants (31, 43, 65). The transgenic lines were numbered as follows: The first number (e.g., 17) refers to the construct used for transformation and the background used (e.g., *tcpQ* or WT), the second number refers to independent lines (i.e., 17-1 and 17-2 are two independent transgenic lines). The third number refers to the homozygous line identified in T3.

A detailed description of the methods used for plasmid construction, protein–protein interaction, protein extraction and detection, RNA extraction and gene expression analyses, anthocyanin content measurements, and flowering time analysis can be found in *SI Appendix*, *Materials and Methods*.

## Supplementary Material

Appendix 01 (PDF)

## Data Availability

All study data are included in the article and/or *SI Appendix*.

## References

[1] I. Paik, E. Huq, Plant photoreceptors: Multi-functional sensory proteins and their signaling networks. Semin. Cell Dev. Biol. **92**, 114–121 (2019). 30946988 10.1016/j.semcdb.2019.03.007 PMC8630751

[2] M. Heijde, R. Ulm, UV-B photoreceptor-mediated signalling in plants. Trends Plant Sci. **17**, 230–237 (2012). 22326562 10.1016/j.tplants.2012.01.007

[3] M. Legris, Y. Ç. Ince, C. Fankhauser, Molecular mechanisms underlying phytochrome-controlled morphogenesis in plants. Nat. Commun. **10**, 5219 (2019). 31745087 10.1038/s41467-019-13045-0 PMC6864062

[4] Q. Wang, C. Lin, Mechanisms of cryptochrome-mediated photoresponses in plants. Annu. Rev. Plant Biol. **71**, 103–129 (2020). 32169020 10.1146/annurev-arplant-050718-100300 PMC7428154

[5] X. D. Lu , Red-light-dependent interaction of phyB with SPA1 promotes COP1-SPA1 dissociation and photomorphogenic development in *Arabidopsis*. Mol. Plant. **8**, 467–478 (2015). 25744387 10.1016/j.molp.2014.11.025

[6] K. Lau, R. Podolec, R. Chappuis, R. Ulm, M. Hothorn, Plant photoreceptors and their signaling components compete for COP1 binding via VP peptide motifs. EMBO J. **38**, e102140 (2019). 31304983 10.15252/embj.2019102140 PMC6745501

[7] O. S. Lau, X. W. Deng, The photomorphogenic repressors COP1 and DET1: 20 years later. Trends Plant Sci. **17**, 584–593 (2012). 22705257 10.1016/j.tplants.2012.05.004

[8] J. Ponnu, U. Hoecker, Illuminating the COP1/SPA ubiquitin ligase: Fresh insights into its structure and functions during plant photomorphogenesis. Front. Plant Sci. **12**, 662793 (2021). 33841486 10.3389/fpls.2021.662793 PMC8024647

[9] X. Han, X. Huang, X. W. Deng, The photomorphogenic central repressor COP1: Conservation and functional diversification during evolution. Plant Commun. **1**, 100044 (2020). 33367240 10.1016/j.xplc.2020.100044 PMC7748024

[10] S. Laubinger, K. Fittinghoff, U. Hoecker, The SPA quartet: A family of WD-repeat proteins with a central role in suppression of photomorphogenesis in *Arabidopsis*. Plant Cell **16**, 2293–2306 (2004). 15308756 10.1105/tpc.104.024216 PMC520934

[11] D. Zhu , Biochemical characterization of *Arabidopsis* complexes containing CONSTITUTIVELY PHOTOMORPHOGENIC1 and SUPPRESSOR OF PHYA proteins in light control of plant development. Plant Cell **20**, 2307–2323 (2008). 18812498 10.1105/tpc.107.056580 PMC2570740

[12] N. Ordoñez-Herrera , A *cop1 spa* mutant deficient in COP1 and SPA proteins reveals partial co-action of COP1 and SPA during *Arabidopsis* post-embryonic development and photomorphogenesis. Mol. Plant. **8**, 479–481 (2015). 25667004 10.1016/j.molp.2014.11.026

[13] K. U. Torii, T. W. McNellis, X. W. Deng, Functional dissection of *Arabidopsis* COP1 reveals specific roles of its three structural modules in light control of seedling development. EMBO J. **17**, 5577–5587 (1998). 9755158 10.1093/emboj/17.19.5577 PMC1170886

[14] M. Holm, C. S. Hardtke, R. Gaudet, X. W. Deng, Identification of a structural motif that confers specific interaction with the WD40 repeat domain of *Arabidopsis* COP1. EMBO J. **20**, 118–127 (2001). 11226162 10.1093/emboj/20.1.118 PMC140188

[15] S. Uljon , Structural basis for substrate selectivity of the E3 ligase COP1. Structure **24**, 687–696 (2016). 27041596 10.1016/j.str.2016.03.002 PMC4856590

[16] J. Ponnu, T. Riedel, E. Penner, A. Schrader, U. Hoecker, Cryptochrome 2 competes with COP1 substrates to repress COP1 ubiquitin ligase activity during *Arabidopsis* photomorphogenesis. Proc. Natl. Acad. Sci. U.S.A. **116**, 27133–27141 (2019). 31822614 10.1073/pnas.1909181116 PMC6936435

[17] L. Trimborn , Cryptochrome 1 promotes photomorphogenesis in Arabidopsis by displacing substrates from the COP1 ubiquitin ligase. Plant J. **121**, e70071 (2025), 10.1111/tpj.70071. 40052249 PMC11886768

[18] P. Cubas, N. Lauter, J. Doebley, E. Coen, The TCP domain: A motif found in proteins regulating plant growth and development. Plant J. **18**, 215–222 (1999). 10363373 10.1046/j.1365-313x.1999.00444.x

[19] S. Kosugi, Y. Ohashi, DNA binding and dimerization specificity and potential targets for the TCP protein family. Plant J. **30**, 337–348 (2002). 12000681 10.1046/j.1365-313x.2002.01294.x

[20] P. Aggarwal , Identification of specific DNA binding residues in the TCP family of transcription factors in *Arabidopsis*. Plant Cell **22**, 1174–1189 (2010). 20363772 10.1105/tpc.109.066647 PMC2879757

[21] Y. Zhang , DNA-TCP complex structures reveal a unique recognition mechanism for TCP transcription factor families. Nucleic Acids Res. **51**, 434–448 (2022). 10.1093/nar/gkac1171 PMC9841405 36546761

[22] M. Nicolas, P. Cubas, TCP factors: New kids on the signaling block. Curr. Opin. Plant Biol. **33**, 33–41 (2016). 27310029 10.1016/j.pbi.2016.05.006

[23] S. Danisman , Analysis of functional redundancies within the *Arabidopsis* TCP transcription factor family. J. Exp. Bot. **64**, 5673–5685 (2013). 24129704 10.1093/jxb/ert337 PMC3871820

[24] T. Koyama, F. Sato, M. Ohme-Takagi, Roles of miR319 and TCP transcription factors in leaf development. Plant Physiol. **175**, 874–885 (2017). 28842549 10.1104/pp.17.00732 PMC5619901

[25] M. J. Mazur , Arabidopsis TCP transcription factors interact with the SUMO conjugating machinery in nuclear foci. Front. Plant Sci. **8**, 2043 (2017). 29250092 10.3389/fpls.2017.02043 PMC5714883

[26] Y. C. Peng , The ubiquitin receptors DA1, DAR1, and DAR2 redundantly regulate endoreduplication by modulating the stability of TCP14/15 in *Arabidopsis*. Plant Cell **27**, 649–662 (2015). 25757472 10.1105/tpc.114.132274 PMC4558651

[27] W. Zhang , The MPK8-TCP14 pathway promotes seed germination in *Arabidopsis*. Plant J. **100**, 677–692 (2019). 31325184 10.1111/tpj.14461

[28] M. Bemer, A. D. J. van Dijk, R. G. H. Immink, G. C. Angenent, Cross-Family transcription factor interactions: An additional layer of gene regulation. Trends Plant Sci. **22**, 66–80 (2017). 27814969 10.1016/j.tplants.2016.10.007

[29] N. Dhaka, V. Bhardwaj, M. K. Sharma, R. Sharma, Evolving tale of TCPs: New paradigms and old lacunae. Front. Plant Sci. **8**, 479 (2017). 28421104 10.3389/fpls.2017.00479 PMC5376618

[30] V. Gastaldi , Class I TCP transcription factors TCP14 and TCP15 promote axillary branching in *Arabidopsis* by counteracting the action of Class II TCP BRANCHED1. New Phytol. **243**, 1810–1822 (2024). 38970467 10.1111/nph.19950

[31] A. Kubota , TCP4-dependent induction of CONSTANS transcription requires GIGANTEA in photoperiodic flowering in Arabidopsis. PLoS Genet. **13**, e1006856 (2017). 28628608 10.1371/journal.pgen.1006856 PMC5495492

[32] J. Liu , MicroRNA319-regulated TCPs interact with FBHs and PFT1 to activate *CO* transcription and control flowering time in Arabidopsis. PLoS Genet. **13**, e1006833 (2017). 28558040 10.1371/journal.pgen.1006833 PMC5469495

[33] X. Li , TCP7 interacts with nuclear Factor-Ys to promote flowering by directly regulating *SOC1* in *Arabidopsis*. Plant J. **108**, 1493–1506 (2021). 34607390 10.1111/tpj.15524

[34] S. Li, S. Zachgo, TCP3 interacts with R2R3-MYB proteins, promotes flavonoid biosynthesis and negatively regulates the auxin response in *Arabidopsis thaliana*. Plant J. **76**, 901–913 (2013). 24118612 10.1111/tpj.12348

[35] T. Narumi , *Arabidopsis* chimeric TCP3 repressor produces novel floral traits in *Torenia fournieri* and *Chrysanthemum morifolium*. Plant Biotechnol.-NAR **28**, 131–140 (2011).

[36] C. Li , UVR8-TCP4-LOX2 module regulates UV-B tolerance in *Arabidopsis*. J. Integr. Plant Biol. **66**, 897–908 (2024). 38506424 10.1111/jipb.13648

[37] I. L. Viola, A. Camoirano, D. H. Gonzalez, Redox-dependent modulation of anthocyanin biosynthesis by the TCP transcription factor TCP15 during exposure to high light intensity conditions in *Arabidopsis*. Plant Physiol. **170**, 74–85 (2015). 26574599 10.1104/pp.15.01016 PMC4704573

[38] J. Dong , The transcription factors TCP4 and PIF3 antagonistically regulate organ-specific light induction of *SAUR* genes to modulate cotyledon opening during de-etiolation in *Arabidopsis*. Plant cell **31**, 1155–1170 (2019). 30914467 10.1105/tpc.18.00803 PMC6533013

[39] A. L. Alem , TCP15 interacts with GOLDEN2-LIKE 1 to control cotyledon opening in *Arabidopsis*. Plant J. **110**, 748–763 (2022). 35132717 10.1111/tpj.15701

[40] J. Paz-Ares, Regia Consortium, REGIA, an EU project on functional genomics of transcription factors from *Arabidopsis thaliana*. Comp. Funct. Genomics **3**, 102–108 (2002). 18628849 10.1002/cfg.146 PMC2447270

[41] G. Castrillo , Speeding *cis*-Trans regulation discovery by phylogenomic analyses coupled with screenings of an arrayed library of *Arabidopsis* transcription factors. PLoS ONE **6**, e21524 (2011). 21738689 10.1371/journal.pone.0021524 PMC3124521

[42] J. F. Palatnik , Control of leaf morphogenesis by microRNAs. Nature **425**, 257–263 (2003). 12931144 10.1038/nature01958

[43] T. Koyama, M. Furutani, M. Tasaka, M. Ohme-Takagi, TCP transcription factors control the morphology of shoot lateral organs via negative regulation of the expression of boundary-specific genes in *Arabidopsis*. Plant Cell **19**, 473–484 (2006). 10.1105/tpc.106.044792 PMC1867346 17307931

[44] A. Maier , Light and the E3 ubiquitin ligase COP1/SPA control the protein stability of the MYB transcription factors PAP1 and PAP2 involved in anthocyanin accumulation in *Arabidopsis*. Plant J. **74**, 638–651 (2013). 23425305 10.1111/tpj.12153

[45] X. W. Deng, P. H. Quail, Genetic and phenotypic characterization of *cop1* mutants of *Arabidopsis thaliana*. Plant J. **2**, 83–95 (1992).

[46] R. P. Hellens , Transient expression vectors for functional genomics, quantification of promoter activity and RNA silencing in plants. Plant Methods **1**, 13 (2005). 16359558 10.1186/1746-4811-1-13 PMC1334188

[47] Z. M. He, X. Y. Zhao, F. N. Kong, Z. C. Zuo, X. M. Liu, TCP2 positively regulates HY5/HYH and photomorphogenesis in *Arabidopsis*. J. Exp. Bot. **67**, 775–785 (2015). 26596765 10.1093/jxb/erv495 PMC4737077

[48] Z. M. He , Identification of a consensus DNA-binding site for the TCP domain transcription factor TCP2 and its important roles in the growth and development of *Arabidopsis*. Mol. Biol. Rep. **48**, 2223–2233 (2021). 33689093 10.1007/s11033-021-06233-z

[49] T. W. McNellis , Genetic and molecular analysis of an allelic series of *cop1* mutants suggests functional roles for the multiple protein domains. Plant Cell **6**, 487–500 (1994). 8205001 10.1105/tpc.6.4.487 PMC160452

[50] S. Jang , *Arabidopsis* COP1 shapes the temporal pattern of CO accumulation conferring a photoperiodic flowering response. EMBO J. **27**, 1277–1288 (2008). 18388858 10.1038/emboj.2008.68 PMC2291449

[51] C. Li , Mutual upregulation of HY5 and TZP in mediating phytochrome A signaling. Plant Cell **34**, 633–654 (2021). 10.1093/plcell/koab254 PMC8774092 34741605

[52] S. H. Hong , CRY1 inhibits COP1-mediated degradation of BIT1, a MYB transcription factor, to activate blue light-dependent gene expression in *Arabidopsis*. Plant J. **55**, 361–371 (2008). 18397371 10.1111/j.1365-313X.2008.03508.x

[53] H. Shi , Seedlings transduce the depth and mechanical pressure of covering soil using COP1 and ethylene to regulate EBF1/EBF2 for soil emergence. Curr. Biol. **26**, 139–149 (2016). 26748855 10.1016/j.cub.2015.11.053 PMC5108888

[54] Y.-S. Hur , *Arabidopsis* transcription factor TCP13 promotes shade avoidance syndrome-like responses by directly targeting a subset of shade-responsive gene promoters. J. Exp. Bot. **75**, 241–257 (2023). 10.1093/jxb/erad402 37824096

[55] Y. Zhou , TCP transcription factors associate with PHYTOCHROME INTERACTING FACTOR 4 and CRYPTOCHROME 1 to regulate thermomorphogenesis in *Arabidopsis thaliana*. iScience **15**, 600–610 (2019). 31078553 10.1016/j.isci.2019.04.002 PMC6547012

[56] X. Han , *Arabidopsis* transcription factor TCP5 controls plant thermomorphogenesis by positively regulating PIF4 activity. iScience **15**, 611–622 (2019). 31078552 10.1016/j.isci.2019.04.005 PMC6548983

[57] A. Gonzalez, M. Zhao, J. M. Leavitt, A. M. Lloyd, Regulation of the anthocyanin biosynthetic pathway by the TTG1/bHLH/Myb transcriptional complex in *Arabidopsis* seedlings. Plant J. **53**, 814–827 (2008). 18036197 10.1111/j.1365-313X.2007.03373.x

[58] W. J. Xu, C. Dubos, L. Lepiniec, Transcriptional control of flavonoid biosynthesis by MYB-bHLH-WDR complexes. Trends Plant Sci. **20**, 176–185 (2015). 25577424 10.1016/j.tplants.2014.12.001

[59] J. P. An , Dynamic regulation of anthocyanin biosynthesis at different light intensities by the BT2-TCP46-MYB1 module in apple. J. Exp. Bot. **71**, 3094–3109 (2020). 31996900 10.1093/jxb/eraa056 PMC7475178

[60] D. B. Li , *Arabidopsis* class II TCP transcription factors integrate with the FT-FD module to control flowering. Plant Physiol. **181**, 97–111 (2019). 31235561 10.1104/pp.19.00252 PMC6716235

[61] I. L. Viola, D. H. Gonzalez, TCP transcription factors in plant reproductive development: Juggling multiple roles. Biomolecules **13**, 750 (2023). 37238620 10.3390/biom13050750 PMC10216028

[62] A. Camoirano, A. L. Alem, D. H. Gonzalez, I. L. Viola, The N-terminal region located upstream of the TCP domain is responsible for the antagonistic action of the *Arabidopsis thaliana* TCP8 and TCP23 transcription factors on flowering time. Plant Sci. **328**, 111571 (2023). 36535527 10.1016/j.plantsci.2022.111571

[63] S. Laubinger , Arabidopsis SPA proteins regulate photoperiodic flowering and interact with the floral inducer CONSTANS to regulate its stability. Development **133**, 3213–3222 (2006). 16854975 10.1242/dev.02481

[64] L. J. Liu , COP1-mediated ubiquitination of CONSTANS Is implicated in cryptochrome regulation of flowering in *Arabidopsis*. Plant Cell **20**, 292–306 (2008). 18296627 10.1105/tpc.107.057281 PMC2276438

[65] T. L. Shimada, T. Shimada, I. Hara-Nishimura, A rapid and nondestructive screenable marker, FAST, for identifying transformed seeds of *Arabidopsis thaliana*. Plant J. **61**, 519–528 (2010). 19891705 10.1111/j.1365-313X.2009.04060.x

