# Supplementary material for: TCP3 is a substrate of the COP1/SPA ubiquitin ligase to regulate anthocyanin accumulation and flowering time in Arabidopsis
Source: Proc Natl Acad Sci U S A. 2025 May 13;122(20):e2426423122. doi: 10.1073/pnas.2426423122 (PMC12107181; doi:10.1073/pnas.2426423122)
Supplement: Supplementary file 1 — Appendix 01 (PDF) [file pnas.2426423122.sapp.pdf]

Supporting Information for

TCP3 is a substrate of the COP1/SPA ubiquitin ligase to regulate anthocyanin accumulation and flowering time in Arabidopsis

Authors and Affiliation: Ruiyan Tao[a,b,1], Ira Trivedi[a], Laura Trimborn[a], Jathish Ponnu[a,c], Blanka Violetta Tóth[a], Ute Hoecker[a,1]

[a] Institute for Plant Sciences and Cluster of Excellence on Plant Sciences (CEPLAS), Department of Biology, Biocenter, University of Cologne, Zülpicher Str. 47b, 50674 Cologne, Germany

[b] Current address: National Key Laboratory of Tropical Crop Breeding, Shenzhen Branch, Guangdong Laboratory for Lingnan Modern Agriculture, Genome Analysis Laboratory of the Ministry of Agriculture, Agricultural Genomics Institute at Shenzhen, Chinese Academy of Agricultural Sciences, Shenzhen 518000, China

[c] Current address: Molecular Cell Biology, Botanical Institute, Department of Biology, Karlsruhe Institute of Technology, Fritz-Haber-Weg 4, 76131 Karlsruhe, Germany.

[1] To whom correspondence should be addressed.

Email: hoeckeru@uni-koeln.de
taoruiyan93@gmail.com

ORCID IDs:

Ruiyan Tao-0000-0003-1072-1867

Jathish Ponnu - 0000-0002-3276-7068

Ute Hoecker - 0000-0002-5636-9777

This PDF file includes:
Supporting text
Figures S1 to S16
Table S1

Supporting text

Supplementary Materials and Methods. Detailed procedures for plasmid construction, protein-protein interactions, protein extraction and immunodetection, RNA extraction and RT-qPCR studies, anthocyanin content quantification and flowering time analysis used in this paper.

Cloning of the constructs used in this study

The primers used for cloning are listed in SI-Appendix, Table S1.

A. Entry plasmid constructions

To construct the TCP3-pEntry3C, TCP4-pEntry3C andTCP10-pEntry3C plasmids, we illustrate the cloning process using TCP3 as an example. The primers TCP3-pEntry3c-F2/ TCP3-pENTRY3C-R2 were used to amplify the open reading frame (CDS) of TCP3 from cDNA derived from Col-0. The PCR product was inserted into the pEntry3c vector, which had been previously digested with XhoI/BamHI, using Gibson assembly. Similarly, the cloning primers for TCP4 were TCP4-pEntry3c-F2/TCP4-pEntry3c-R2 and the cloning primers for TCP10 were TCP10-pEntry3c-F2/TCP10-pEntry3c-R2. Besides, we amplified fragments TCP3-D1 (expressing 1-114 aa, TCP3-D1-pEntry3c-F/TCP3-D1-pEntry3c-R), TCP3-D2 (115-391 aa, TCP3-D2-pEntry3c-F/TCP3-D2-pEntry3c-R), TCP3-D3 (49-114 aa, TCP3-D3-pEntry3c-F/TCP3-D3-pEntry3c-R), and TCP3-D4 (expressing 256-391 aa, TCP3-D4-pEntry3c-F/TCP3-D4-pEntry3c-R). These fragments were inserted into the pEntry3C vector (XhoI/BamHI), resulting in the constructs TCP3-D1-pEntry3C, TCP3-D2-pEntry3C, TCP3-D3-pEntry3C, and TCP3-D4-pEntry3C.

For constructing the miRNA319-resistant rTCP3-pEntry3C plasmid, we employed overlap extension polymerase chain reaction (OE-PCR). The primers TCP3-pEntry3c-F2/TCP3 MutJAW-R1 and TCP3-MutJAW-F1/TCP3-pEntry3c-R2 were used to clone the 5' and 3' parts of the TCP3 CDS, respectively. The junction of these segments corresponded to the recognition site of miRNA319. The recovered PCR products were mixed in a 1:1 ratio and used as a template for a subsequent PCR with the primers

TCP3-pEntry3c-F2 and TCP3-pEntry3c-R2. The final PCR products were inserted into the pEntry3C vector using Gibson assembly.

Similarly, to construct the TCP10-AA-pEntry3C plasmid, the primers TCP10-pEntry3c-F2/TCP10-AAmut-R2 and TCP10-AAmut-F2/TCP10-pEntry3c-R2 were used to clone two parts of TCP10 respectively. The full-length of TCP10-AA-pEntry3c was cloned by TCP10-pEntry3c-F1/TCP10-pEntry3c-R1.

To construct NLS-GUS-pEntry3C vector, the CDS of GUS was amplified using the primers NLS-GUS-Gib-fwd/NLS-GUS-Gib-rev. The PCR products were inserted into the pEntry3C vector (XhoI/BamHI) using Gibson assembly.

B. Plasmids for Y2H and Y3H assays

In Y2H assays, we chose pACT2-GW and pAS2-GW as the Gateway-compatible activation domain (AD) and binding domain (BD) vectors, respectively (1). Using LR recombination reactions, we transferred our constructed entry vectors into the pACT2-GW vector. This process resulted in the following constructs: AD-TCP3, AD-TCP4, AD-TCP10, AT-TCP10AA, AD-TCP3-D1, AD-TCP3-D2, AD-TCP3-D3, and AD-TCP3-D4. Constructs such as BD-COP1, BD-SPA1, BD-COP1-N (equal to BD-COP1-RING+CC), BD-SPA1-N (equal to BD-SPA1-N696), BD-COP1-C (equal to BD-NLS-COP1-WD) and BD-SPA1-C (equal to BD-SPA1-WD) were described previously (1, 2).

In Y2H assays shown in Fig S8, COP1, COP1W467A and COP1F595A were constructed by OE-PCR. The COP1-pGBK-F/COP1-W467A-R and COP1-W467A-F/COP1-pGBK-R were used to clone COP1W467A gene; COP1-pGBK-F/COP1-F595A-pGBK-R and COP1-F595A-pGBK-F/COP1-pGBK-R were used to clone COP1F595A gene and COP1-pGBK-F/COP1-pGBK-R were use to clone COP1 gene. The three genes were ligated into pGBKT7 plasmid (630443, Takara, Japan) which was digested by EcoRI/BamHI.

In Y3H experiments, we utilized the pBridge vector (Takara). The CDS of COP1 was cloned by COP1-vazyme-fwd/COP1-vazyme-rev and inserted into pBridge digested by EcoRI and BamHI. The sequenced pBridge-BD-COP1 was digested by NcoI and PstI

and full length of SPA1 and TCP10 was cloned by pBridge-BD-COP1+SPA1-F1/pBridge-BD-COP1+SPA1-R1 and pBridge-BD-COP1+TCP10-F1/pBridge-BD-COP1+TCP10-R1.

C. Plasmids for colocalization and FRET-FLIM assays

pAMARENA (with N-terminal mCherry tagging) (3) and pENSG-YFP (with N-terminal YFP tagging) (4) were used for colocalization and FRET-FLIM experiments. LR reactions were performed by mixing the corresponding entry vectors with the pAMARENA vector to generate mCherry-TCP3, mCherry-TCP3-N1, mCherry-TCP3-N2, mCherry-TCP3-N3, and mCherry-TCP3-N4 plasmids. The constructs YFP-COP1 and YFP-SPA1 have been previously described (5).

D. Plasmids for LCI assays

The pCambia1300-nLUC and pCambia1300-cLUC vectors (6) were used in LCI experiments. The full-length CDS of TCP3 was amplified using the primers TCP3-nLUC-F/TCP3-nLUC-R, and subsequently inserted into the pCambia1300-nLUC vector, which had been digested with SalI and KpnI. The full-length CDS of COP1 and SPA1 was amplified using the primers KpnI-COP1-fwd/SalI-Stop-COP1-rev and KpnI-SPA1-fwd/SalI-Stop-SPA1-rev respectively, and subsequently inserted into the pCambia1300-cLUC vector, which had been digested with KpnI and SalI. YFP-NLS-GUS was constructed by mixing pENSG-YFP vector and NLS-GUS-pEntry3C via LR reaction.

E Plasmids for plant transformations

The constructs rTCP3-pFAST-R05 (C-YFP tag) were respectively derived from the vectors rTCP3-pEntry3c. Each of these constructs was specifically ligated into pFAST-R05 vector by LR reaction (7).

F. Plasmids for Dual-luciferase assays

The pGreen62-SK and pGreen0800-LUC plasmids (8) were digested with BamHI and HindIII. The CDS of TCP3, PAP2, and TT8 were cloned into pGreen62-SK plasmids

using the primers TCP3-pGreen62-SK-F/TCP3-pGreen62-SK-R, PAP2-pGreen62-SK-F1/PAP2-pGreen62-SK-R1, and TT8-pGreen62-SK-F1/TT8-pGreen62-SK-R1, respectively, via Gibson assembly. Additionally, an 842 bp fragment upstream of the ATG of the *DFR* promoter, a 917 bp fragment of the *LDOX* promoter and a 1542 bp fragment of the *CO* promoter were cloned by pGreenII0800-DFRpro-F/pGreenII0800-DFRpro-R, pGreenII0800-LDOX1pro-F/pGreenII0800-LDOX1pro-R and proCO-LUC-F1/ proCO-LUC-R1, respectively. The fragments were inserted into pGreen0800-LUC digested by HindIII and BamHI. The names of the plasmids are TCP3-pGreen62-SK, PAP2-pGreen62-SK, TT8-pGreen62-SK, *proDFR*-pGreen0800-LUC, *proLDOX*-pGreen0800-LUC, *proCO*-pGreen0800-LUC.

G. Plasmids for CRISPR-Cas9

We employed the Golden Gate method for vector construction using shuttle vectors, including pDGE332, pDGE333, pDGE335, and pDGE337, with pDGE652 working as the final transformation vector (9). All single guide RNAs (sgRNAs) were annealed at 98°C for 5 minutes to form double strands. The reaction mixture for assembling shuttle vectors contained 60 ng of shuttle vector, 50 fmol of hybridized oligos, 0.3 µL of *BpiI* (FD1014, ThermoFisher, USA), 0.3 µL of T4 DNA ligase, 1 µL of 10×T4 Ligase buffer (EL0011, ThermoFisher, USA), and H_2O to a final volume of 10 µL. The reaction was performed under the following PCR conditions: 37°C for 2 minutes, 16°C for 5 minutes, repeated for 10-30 cycles, followed by 50°C for 10 minutes and 80°C for 10 minutes. The assembled shuttle vectors were then transferred into the pDGE652 vector using a reaction mixture containing 40 ng of each shuttle vector, 300 ng of pDGE652, 1 µL of *Bsa*I (R3733S, NEB, USA), 2 µL of T4 DNA ligase, 2 µL of 10×T4 Ligase buffer, and water to a final volume of 20 µL, under the same PCR conditions described above.

H. Plasmids for protein purification and pull-down assays

TCP3 was ligated into the pGEX4T-1 vector (GE28-9545-49, Merck, Germany) using the primers TCP3-pGEX4T-1-F1 and TCP3-pGEX4T-1-R1. The pGEX4T-1 vector was pre-digested with EcoR Ⅰ and Not Ⅰ. The COP1-pETG40A was constructed by LR

reaction mixed by pETG40A (A. Geerlof, EMBL) and COP1's entry plasmid. The COP1's entry plasmid was described before (1).

Y2H and Y3H assays

In yeast growth experiments, 500 ng of AD- and 500 ng of BD-based plasmids were introduced into the AH109 yeast strain according to the protocol of the Frozen EZ-yeast transformation kit (T2001, ZYMO Research Company). Following transformation, the yeast colonies were selected on synthetic drop-out (SD) medium lacking leucine (Leu) and tryptophan (Trp), but supplemented with glucose (SD/-Leu/-Trp +Glucose). To induce the interaction and screen for protein-protein interactions, we mixed ten colonies from each transformation and resuspended them in sterile water. These suspensions were then plated on SD medium with OD_{600}=1, 0.1, 0.01 and 0.001, specifically SD/-Leu/-Trp/- Histidine (His) supplemented with glucose and 0.5 mM 3-Amino-1,2,4-triazole (3-AT).

In ONPG assays, combinations of the BD-bridge or BD and AD plasmids were co-transformed into the yeast strain AH109 by the same kit mentioned above. The transformed yeast cells were grown on SD/-Leu/-Trp medium, and then dropped-out onto SD/-Leu/-Trp (Y2H) or SD/-Leu/-Trp/-Met (Y3H) plates. For the quantification assay, yeast cells were harvested from plates and adjusted to OD_{600}=1. After multiple times of liquid nitrogen disruption of cells, we then measured the β-galactosidase activity in these cells using ortho-Nitrophenyl-β-galactoside (ONPG) as the substrate.

Particle bombardment, colocalization and FRET-FLIM assays

Particle bombardment, colocalization and FRET-FLIM assays were performed according to previous reports (2, 5).

Protein purification and pull-down assays

pGEX4T-1, TCP3- pGEX4T-1 and COP1-pETG40A were transferred to E. coli Rosetta strain. Positive colonies were selected and inoculated into LB medium, followed by incubation at 37°C until the OD_{600} reached 0.8-1.2. IPTG was then added to a final

concentration of 0.5 mM, and the cultures were shaken overnight at 22°C. The cultures were collected the next day, and cell disruption was performed using ultrasonication (SONIFIER 250, Branson Ultrasonics, USA), with each cycle lasting 5 minutes, with 6 seconds of sonication followed by 5 seconds of rest, for a total of 4-8 cycles depending on the disruption efficiency. The disrupted cells were centrifuged at 4°C, and the supernatant was collected.

Glutathione Agarose (sc-2009, Santa Cruz Biotechnology, USA) was added to the supernatants of pGEX4T-1 and TCP3-pGEX4T-1, while Amylose Resin (E8021S, NEB, USA) was added to the supernatant of COP1-pMALc5x, and the mixtures were incubated at 4°C for 2-3 hours to allow binding.

For GST-tagged proteins, nonspecifically bound proteins were washed off with 1× PBS (137 mM NaCl, 2.7 mM KCl, 10 mM Na_2HPO_4, 1.8 mM KH_2PO_4, pH 7.4), and GST-tagged proteins were eluted using 50 mM GSH dissolved in 1× PBS. For MBP-tagged proteins, nonspecifically bound proteins were washed off with MBP column binding buffer (20 mM Tris-HCl, 200 mM NaCl, 1 mM EDTA, pH 7.5).

The MBP-COP1 protein bound to amylose was separately incubated with 10 μg of GST-TCP3 protein or 10 μg of GST protein. An additional 10 μg of GST-TCP3 protein was incubated with amylose alone as a control. A portion of each mixture was reserved as the Input, while the remaining mixtures were incubated at 4°C for 2 hours. After the reaction, the samples were eluted using MBP Elution Buffer (10 mM maltose dissolved in MBP column binding buffer).

For protein detection, proteins were resolved on a 10% polyacrylamide SDS gel and transferred onto a PVDF membrane using the wet blotting method. The membrane was blocked with Roti-Block (Carl Roth, Karlsruhe, Germany) for 1 h at room temperature. Detection of GST and MBP was performed using anti-GST (MA4-004, ThermoFisher, USA; 1:2000 diluted) and anti-MBP (E8032S, NEB, USA; 1:5000 diluted) antibody, respectively. Secondary antibodies included anti-mouse-HRP (Sigma-Aldrich, St. Louis, USA; 1:50000 diluted).

LCI and dual-luciferase assays

Tobacco transient expression assays were performed by *Agrobacterium tumefaciens* infiltration of *N. benthamiana* leaves. All relevant plasmids were transformed into *A. tumefaciens* strain GV3101. Overnight cultures of *A. tumefaciens* were harvested by centrifugation and resuspended in MES buffer (10 mM MES, pH=5.8 10 mM $MgCl_2$, 0.2 mM Acetosyringone) to a final concentration of OD_{600}=1.2-1.6. To prevent gene silencing, RK19 was co-infiltrated. Following infiltration, the tobacco plants were incubated in darkness for 12 h, then kept under long-day conditions for 3-4 days.

The LCI assays were performed according to (10). For the protein interaction assays between TCP3-nLUC and cLUC-COP1 or cLUC-SPA1, the nLUC and cLUC-containing Agrobacterium cultures were mixed in a 1:1 ratio. For assays assessing the interaction of TCP3-nLUC and cLUC-COP1 in the presence or absence of YFP-SPA1, nLUC-, cLUC-, and YFP-containing Agrobacterium cultures were mixed in a 1:1:1 ratio. The luciferase activity was imaged by the ImageQuant™ LAS 4000 mini (GE Healthcare, Chicago, IL, USA). The YFP signal was quantified by TECAN with the following setting: ex.: 483 nm; em: 535 nm; gain: optimal; the number of flashes: 40.

For dual-luciferase assays, *A. tumefaciens* cultures harboring the pGreen62-SK and pGreen0800-LUC plasmids were mixed in a 10:1 ratio. The activities of the FLUC and RLUC enzymes were analyzed using the Dual-Luciferase® Reporter Assay System (E1910, Promega, Wisconsin, USA) with TECAN. The FLUC and REN activities were analyzed in two independent experiments with four biological replicates for each assay.

Protein extraction and immunodetection

For co-immunoprecipitation assays and *in vivo* ubiquitination assays, protein was extracted from Arabidopsis seedlings using YODA extraction buffer (50 mM Tris, pH 7.5; 150 mM NaCl; 1 mM EDTA; 10% glycerol; 5 mM DTT; 1% protease inhibitor cocktail (Sigma-Aldrich, St. Louis, USA); 10 μM MG132 (ThermoFisher, Waltham, USA); 0.1% Triton X-100). For protein extraction related to protein abundance studies, SDS extraction buffer was used (0.125 M Tris-HCl, pH 6.8; 4% SDS; 20% glycerol; freshly added 1x protease inhibitor cocktail (Sigma-Aldrich, St. Louis, USA) and 1 mM PMSF). Plant materials were flash-frozen in liquid nitrogen, ground to a fine powder,

and homogenized in the respective extraction buffer. The homogenates were centrifuged at 13.000 rpm for 10 minutes at 4 ° C. Protein concentrations were determined using the BCA or Bradford method, and samples were normalized to equal protein concentrations for loading.

Detection of GFP-tagged proteins was performed using an anti-GFP-HRP antibody (130-091-833, Miltenyi Biotec, Germany; 1:5000 diluted). COP1 detection utilized an α-COP1 antibody (Balcerowicz et al. 2011) and equal protein loading was assessed with an anti-α-Tubulin antibody (T5168, Merck, Germany; 1:5000 diluted). Secondary antibodies included anti-mouse-HRP (12-349, Sigma-Aldrich, USA; 1:50000 diluted) and anti-rabbit-HRP (31460, Thermo Fisher, USA; 1:50000 diluted).

Co-immunoprecipitation (Co-IP) and ubiquitination detection in vivo

For Co-IP assays, we utilized rTCP3-GFP/*tcpQ* (line 17-3-1 and line 17-4-3), *tcpQ*, and *cop1-4* seedlings. All seedlings were grown under continuous white light for 7 days. The seedlings were then treated with 50 μM MG132 under vacuum for 15 min before being shifted to darkness for 4h. Protein extraction was performed using YODA buffer, and TCP3-GFP protein was affinity-purified using μMACS™ and MultiMACS™ GFP Isolation Kits (Miltenyi Biotec, Bergisch Gladbach, Germany). The entire experiment was conducted following the instructions provided in the kit manual and the wash buffer and elution buffer were provided by the kit. The TCP3-GFP protein, COP1 and Tubulin were detected using their respective antibodies.

For in vivo ubiquitination detection, Col-0, *cop1-4*, rTCP3-GFP/Col-0 (line 23-5-5), rTCP3-GFP/*cop1-4* (line 23-5-5/*cop1-4*) seedlings were used. All seedlings were grown in white light for 7 days, then treated with 50 μM MG132 and transferred to darkness. The TCP3-GFP protein was isolated with the same kit as for Co-IP assays. The TCP3-GFP, ubiquitin (14-6078-82, ThermoFisher, USA; 1:2500 diluted) and Tubulin proteins were detected by corresponding antibodies.

Protein degradation *in vivo*

For the detection of protein abundance upon light-to-dark transition, two rTCP3-GFP/*tcpQ* lines (line 17-3-1 and line 17-4-3) and *tcpQ* were grown in white light for 7 days. Subsequently, half of the seedlings were transferred to darkness for 12 h, while the remaining half continued to grow under light conditions for an additional 12 h.

For MG132 treatment, 7-day-old rTCP3-GFP/*tcpQ* seedlings (line 17-4-3) grown in white light were transferred from solid to liquid MS medium containing either 50 μM MG132 or 0.5% dimethyl sulfoxide (DMSO) (mock treatment). These seedlings were then vacuum infiltrated for 15 min. After infiltration, the seedlings were shifted to darkness for 0, 8 and 16 h. Protein was extracted using SDS buffer, and TCP3-GFP and TUB were detected using respective antibodies.

RNA extraction, first-strand cDNA synthesis and RT-qPCR analysis

Total RNA was isolated from Arabidopsis seedlings or leaves using the NucleoSpin RNA Plant kit (Macherey-Nagel, Düren, Germany) in accordance with the manufacturer's instructions. 500 ng of RNA was treated with DNaseI to remove contaminating genomic DNA. DNase- and heat-treated RNA was subsequently used for reverse transcription with oligo-dT primers.

RT-qPCR analyses were performed as described previously (5). Primers, cDNA and qPCR Master Mix (Promega, Wisconsin, USA) were mixed. The detection was performed by QuantStudio Real-Time-PCR (Thermo Fisher, Waltham, USA). ACTIN was used as an internal control and relative transcript levels were calculated using the $2^{-\Delta CT}$ method. All primers used for RT-qPCR are listed in Table S1.

Anthocyanin extraction and quantification

Twenty seedlings grown either in 100 μmol m^{-2} s^{-1} Wc or in darkness for 9 days on MS + 3% sucrose were collected. The anthocyanin extraction and quantification were performed according to previous studies (2).

Flowering time analysis

Flowering time was measured according to previous study (5).

Protein sequence alignment

TCP3, TCP4 and TCP10 protein sequences were downloaded from TAIR (https://www.arabidopsis.org/). DNAman (https://www.lynnon.com/) was employed to perform a multiple protein alignment with BLOSUM (Blocks Substitution Matrix).

Statistical analyses

The statistical significance of the results was analyzed by Student's *t* test or ANOVA.

Accession Numbers

TCP3 (AT1G53230), TCP4 (AT3G15030), TCP10 (AT2G31070), SPA1 (AT2G46340), COP1 (AT2G32950), CO (AT5G15840), FT (AT1G65480), PAP1 (AT1G56650), PAP2 (AT1G66390), TT8 (AT4G09820), CHS (AT5G13930), CHI (AT3G55120), DFR (AT5G42800), LDOX (AT4G22880).

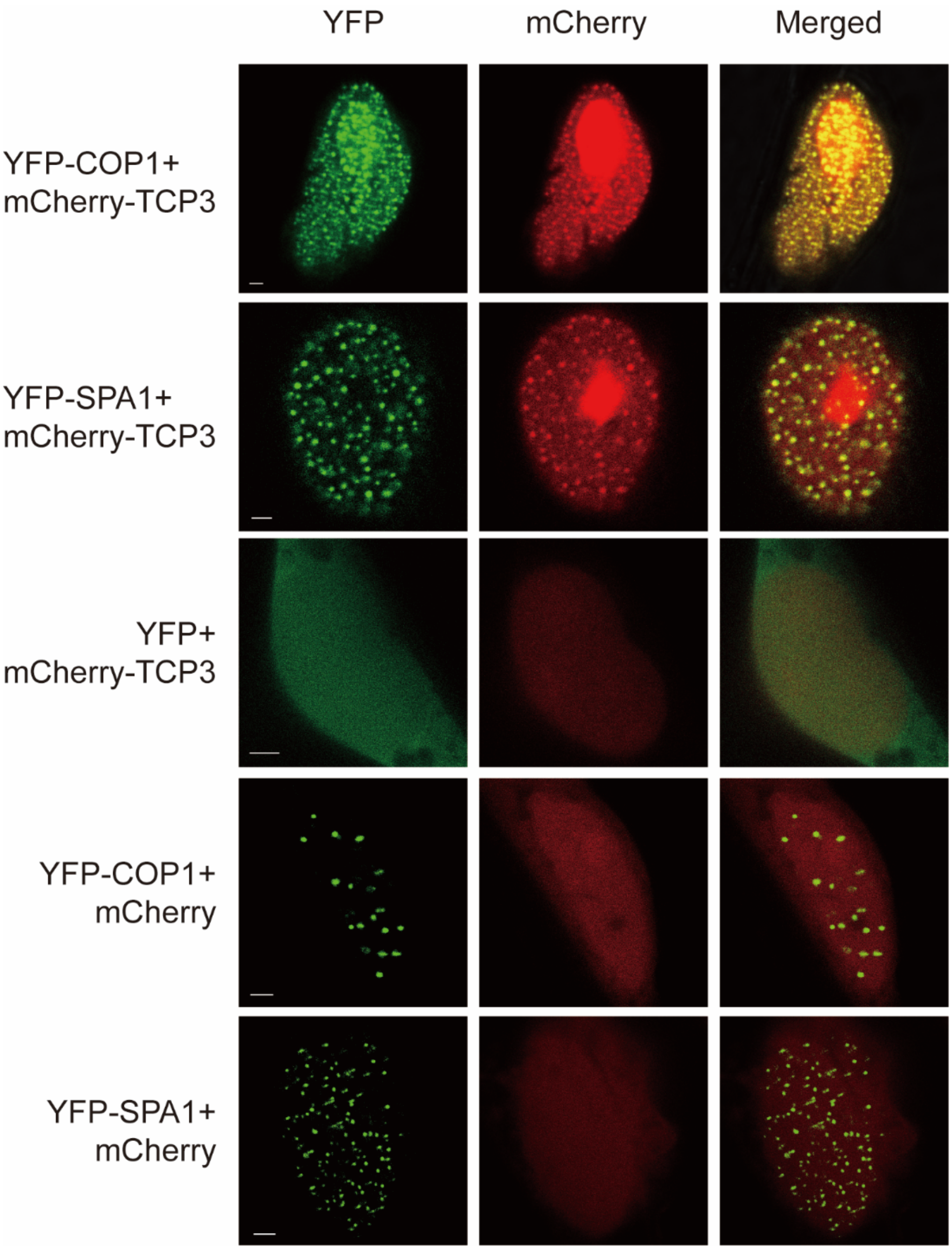

Fig S1: Colocalization analysis in leek epidermal cells co-expressing YFP-COP1 or YFP-SPA1 and mCherry-TCP3 following particle bombardment. The channels were merged to demonstrate colocalization. (Scale bars: 10 μm)

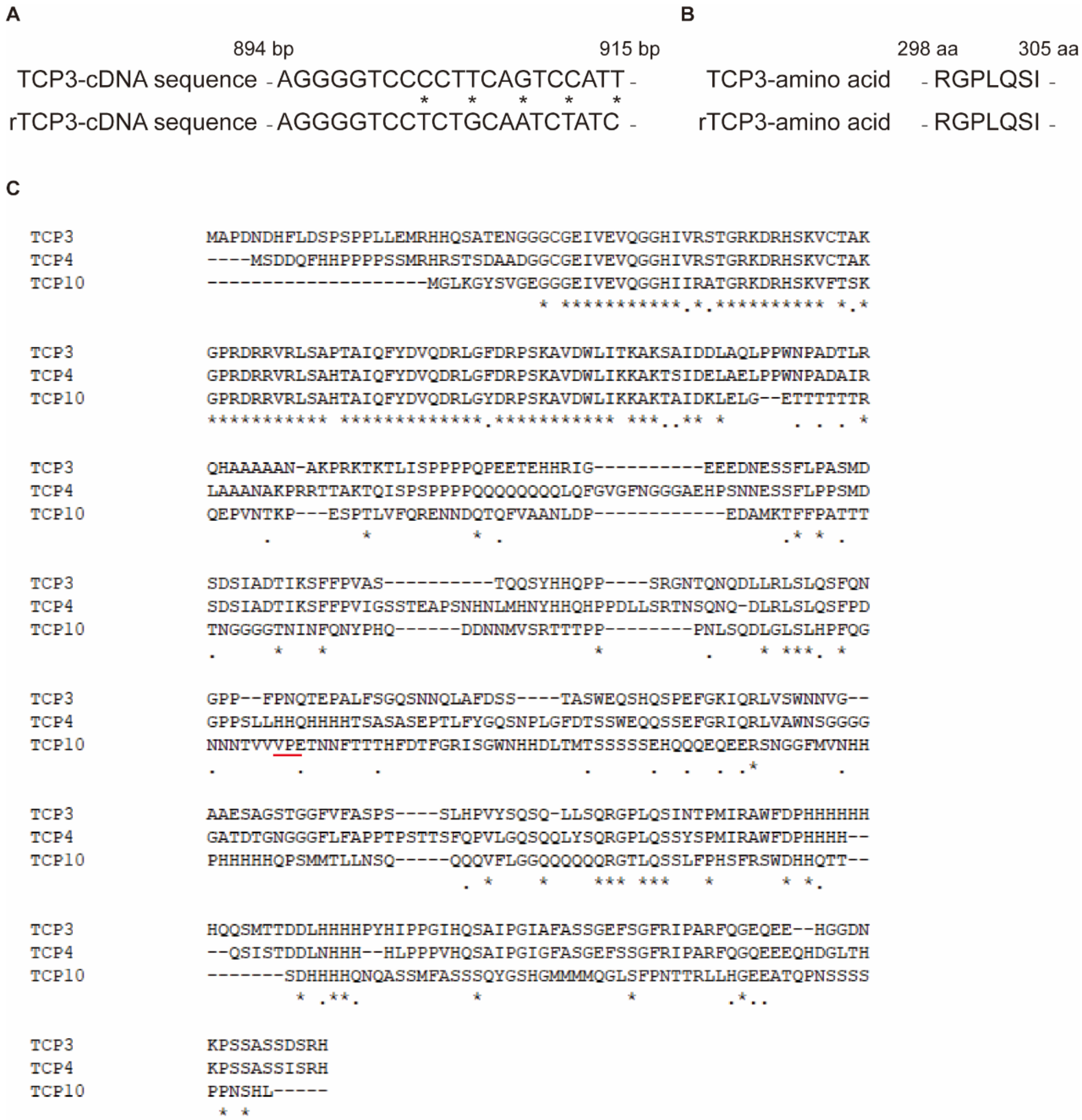

Fig S2: Design of the miR319-resistant *rTCP3* sequence and amino acid sequence alignment of TCPs.

(A-B) cDNA sequence alignment **(A)** and amino acid sequence alignment **(B)** of wild-type TCP3 and rTCP3 altering the miR319 recognition site without changing the amino acid sequence. Asterisks indicate base pair changes in *rTCP3*.

(C) Amino acid sequence alignment of TCP3, TCP4 and TCP10. The red line marks a potential VP motif in TCP10. Asterisks means the same amino acid among three TCPs.

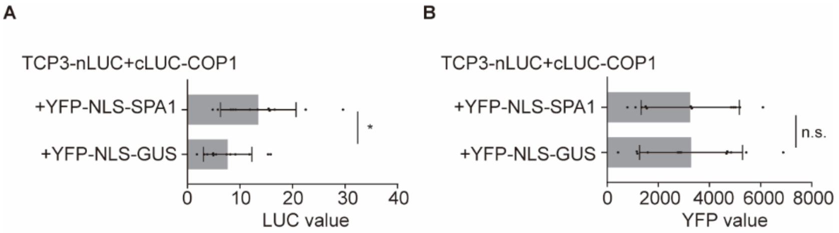

Fig S3: Quantitative analysis of luminescence and fluorescence intensity in tobacco leaves of Fig 1G. Luciferase activities **(A)** and YFP fluorescence **(B)** of 10 transfected leaves were quantified. Asterisks indicate significant differences between the indicated pairs (Student's *t* test, $*p < 0.05$, n.s.=not significant). NLS=nuclear localization sequence.

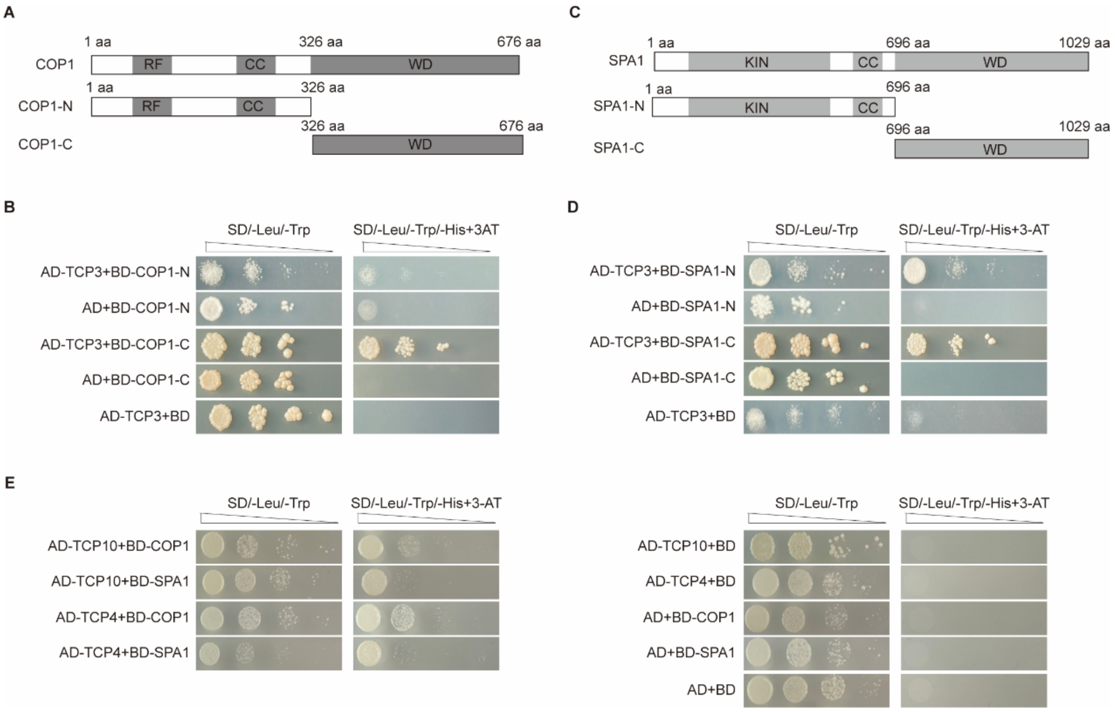

Fig S4: Domain mapping between TCP3 and COP1 or SPA1 and the interaction between TCP4, TCP10 and COP1 or SPA1.

(A, C) Schematic representation of COP1 (A) and SPA1 (C) alongside their deletion-derivatives. RF=RING finger; KIN=kinase domain; CC=coiled-coil domain; WD=WD-repeat domain.

(B, D) Y2H assays investigating the interaction between AD-TCP3 and BD-COP1 (B) or BD-SPA1 (D) and their respective deletion-derivatives.

(E) Y2H assays studying the interaction between AD-TCP4, AD-TCP10 and BD-COP1 or BD-SPA1. Transformed yeast cells in **B**, **D** and **E** were grown on the indicated media at decreasing OD.

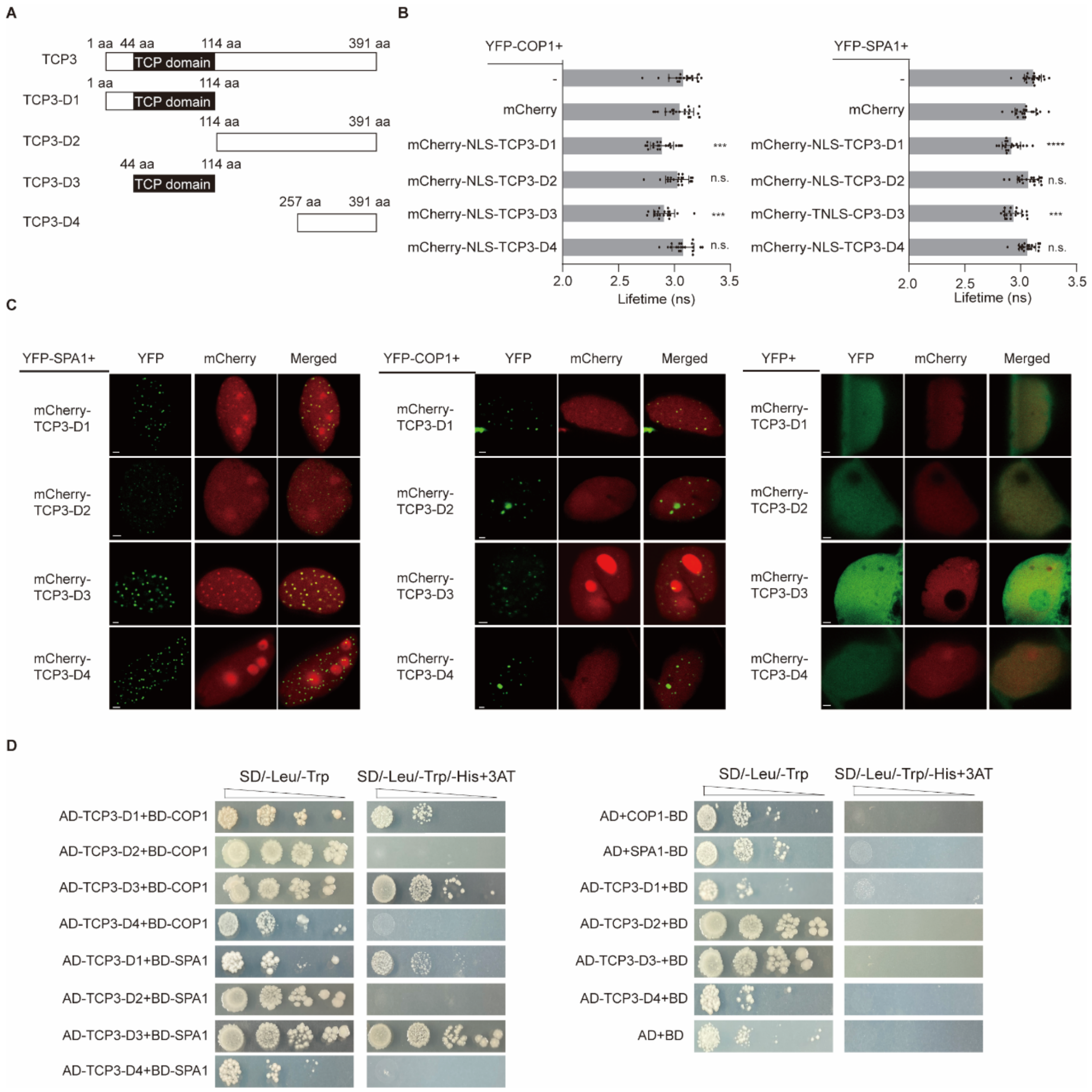

Fig S5: Colocalization analysis of plant cells co-expressing mCherry-TCP3 domains and YFP-COP1 or YFP-SPA1.

(A) Schematic representation of TCP3, COP1 and SPA1 proteins alongside their deletion derivatives. RF=RING finger; CC=coiled-coil domain; WD=WD-repeat domain; KIN=kinase domain.

(B) FRET-FLIM analysis of leek cells transfected with the indicated plasmid combinations using particle bombardment. Error bars indicate the standard deviation of 20 analyzed cells. Asterisks denote significant differences in the respective lifetime compared with YFP-COP1+mCherry or YFP-SPA1+mCherry, respectively (Student's t test, ***p < 0.001, ****p < 0.0001, n.s.=not significant).

(C) Confocal images of leek cells expressing the indicated fusion proteins after particle bombardment. The YFP, mCherry and merged channels are shown. (Scale bars: 10 µm).

(D) Y2H assays investigating the interaction between AD-TCP3 and BD-COP1 or BD-SPA1 and their respective deletion-derivatives. Transformed yeast cells were grown on the indicated media at decreasing OD.

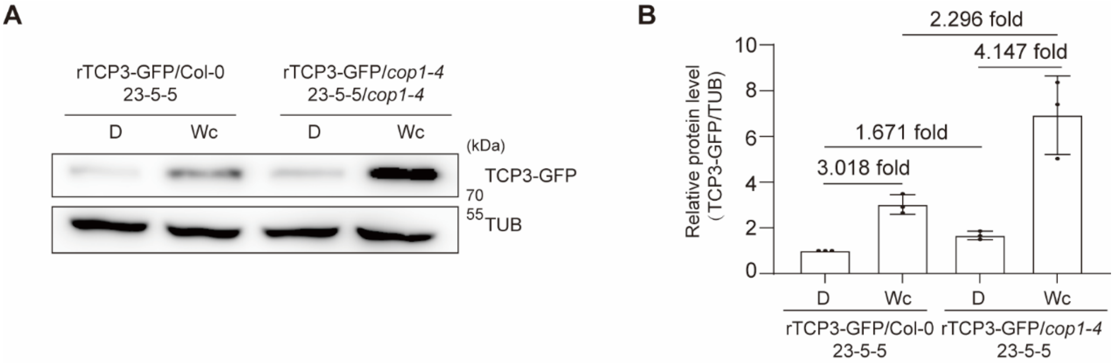

Fig S6: TCP3-GFP protein levels in Col-0 wild-type and *cop1-4* mutant backgrounds in seedlings grown in continuous darkness or Wc.

Seedlings were grown in Wc (100 μmol m^{-2} s^{-1}) or darkness for 7 days. TCP3-GFP and TUB proteins were detected using α-GFP and anti-α-Tubulin antibodies, respectively **(A)**. Quantification of relative rTCP3-GFP/TUB protein levels, with TCP3-GFP/TUB levels in dark-grown rTCP3-GFP/Col-0 23-5-5 seedlings set to 1 **(B)**. Error bars represent the standard deviation from three independent experiments.

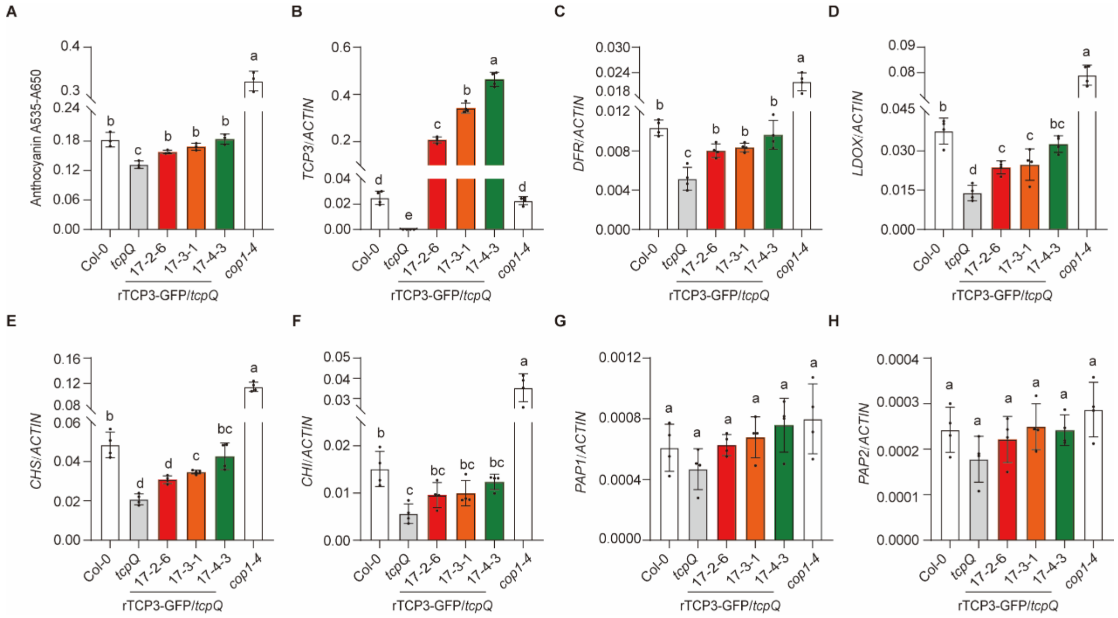

Fig S7: TCP3 increases anthocyanin accumulation in Arabidopsis seedlings grown in Wc.

(A) Anthocyanin content of Arabidopsis seedlings of the indicated genotypes. Seedlings were grown for 9 d in Wc**.**

(B-H) Transcript level analysis of *TCP3*, *CHS*, *CHI, DFR*, *LDOX*, *PAP1* and *PAP2*. Seedlings were grown in Wc for 7 d. Transcript levels were normalized to *ACTIN*. Error bars indicate the standard deviation from four biological replicates. Letters denote significant differences between genotypes; genotypes sharing the same letter do not significantly differ, as determined by one-way ANOVA ($p < 0.05$).

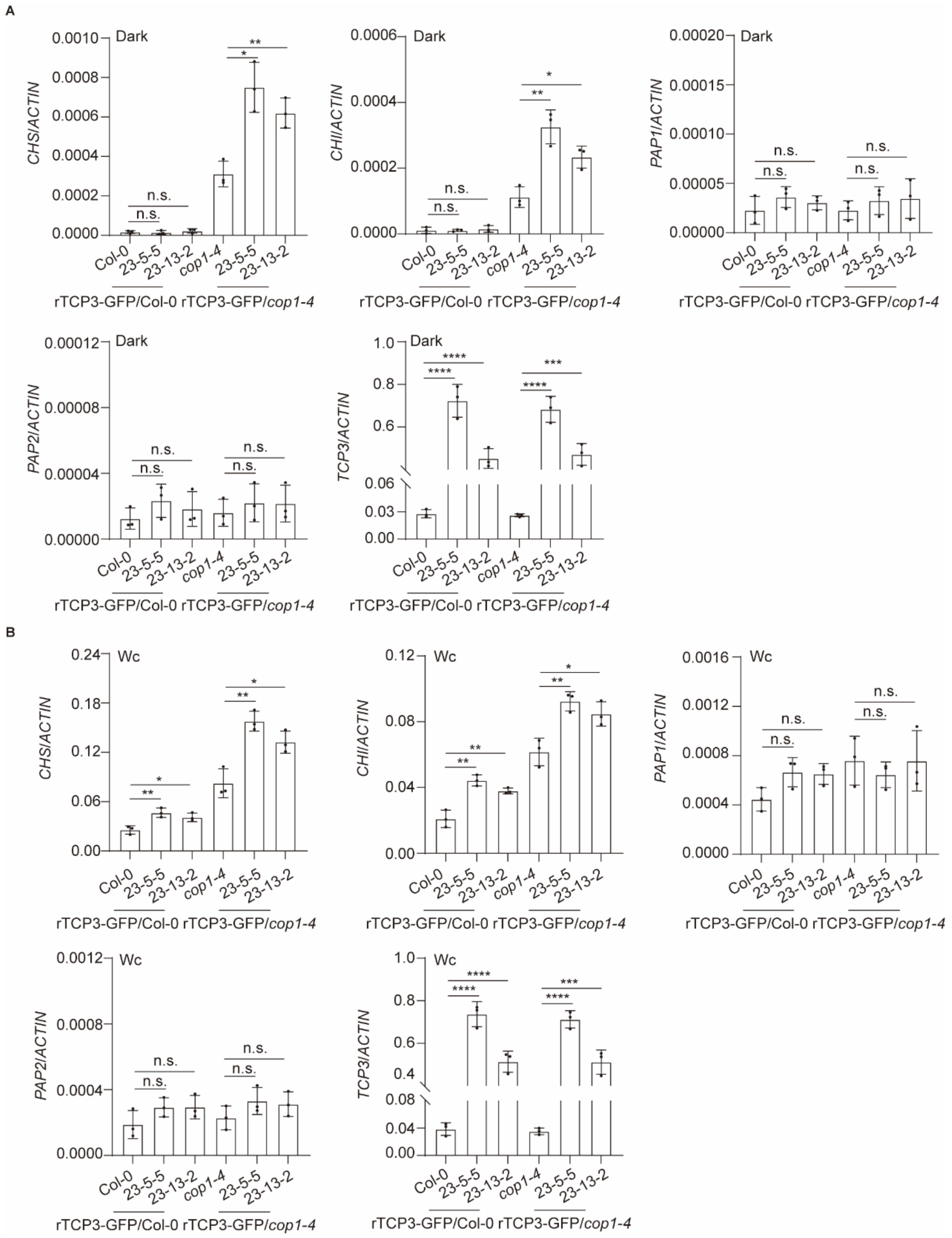

Fig S8: *CHS, CHI, PAP1, PAP2* and *TCP3* transcript levels in seedlings of the indicated genotypes grown in darkness or Wc.

Seedlings were grown for 7 days in darkness **(A)** or Wc **(B)**. Error bars indicate the standard deviation from three biological replicates. Error bars represent the standard deviation from three biological replicates. Asterisks denote significant differences between the indicated pairs (Student's *t* test, ****$p < 0.0001$, ***$p < 0.001$, **$p < 0.01$, *$p < 0.05$).

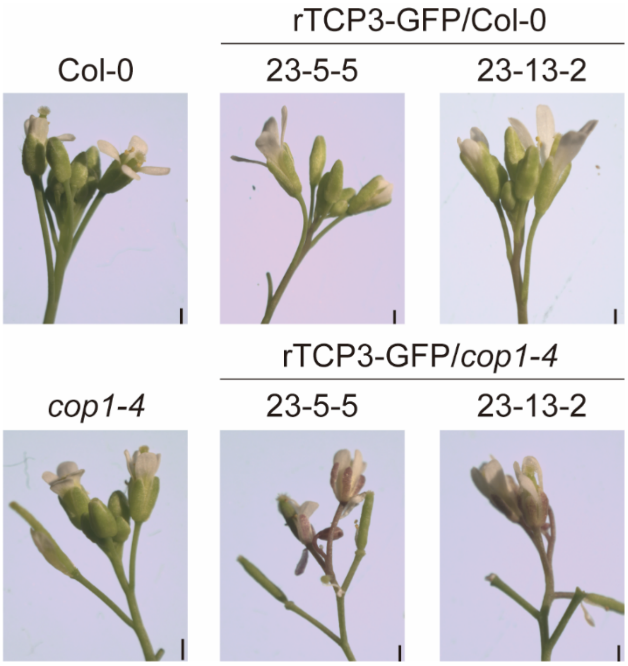

Figure S9: rTCP3-GFP/*cop1-4* plants exhibit purple stem, pedicel and sepal tissues. Col-0 and rTCP3-GFP/Col-0 plants were photographed after 4 weeks in LD, while *cop1-4* and rTCP3-GFP/*cop1-4* plants were photographed after 3 weeks in LD. Scale bar= 1 mm.

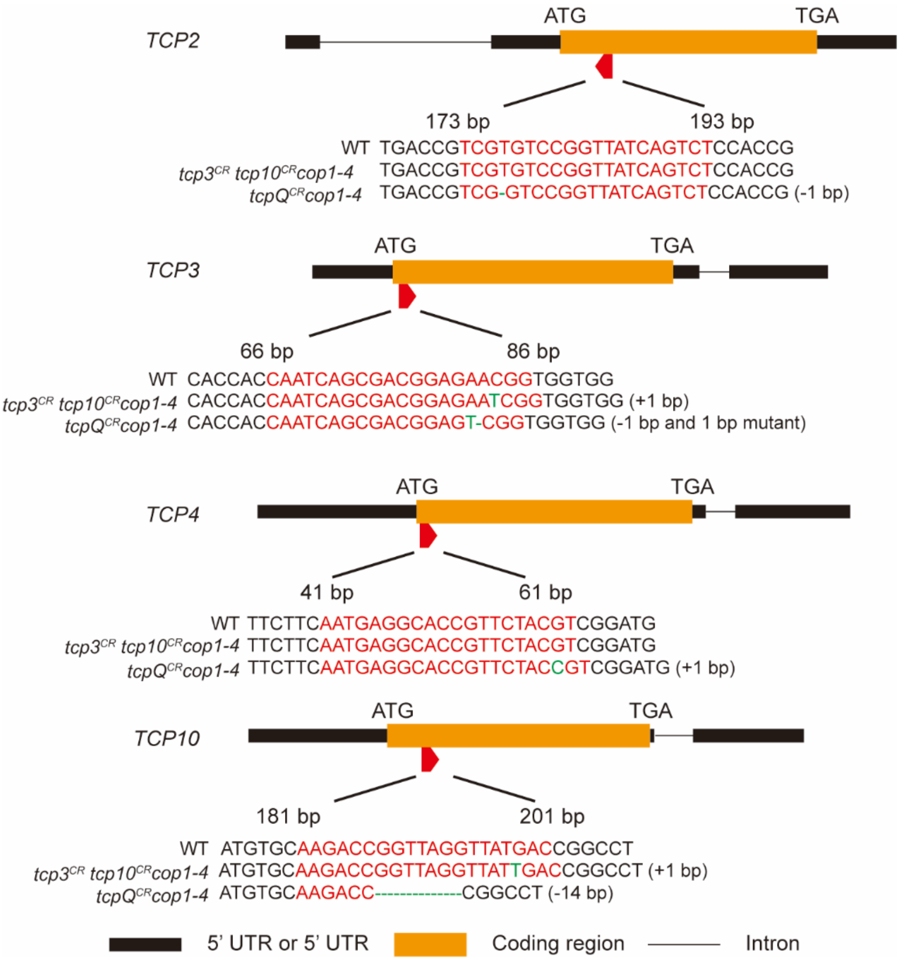

Fig S10: Schematic representation of genotyping of two independent *TCP*-based CRISPR-Cas9 lines. The *tcp3^{CR}tcp10^{CR}cop1-4* line carries mutated *TCP3* and *TCP10* in the *cop1-4* background, while the *tcpQ^{CR}cop1-4* line carries mutated *TCP2*, *TCP3*, *TCP4* and *TCP10* in the *cop1-4* background. The red arrow indicates the direction of the sgRNA from 5' to 3', while the red bases represent the sgRNA sequence. Green bases or green gaps indicate mutations.

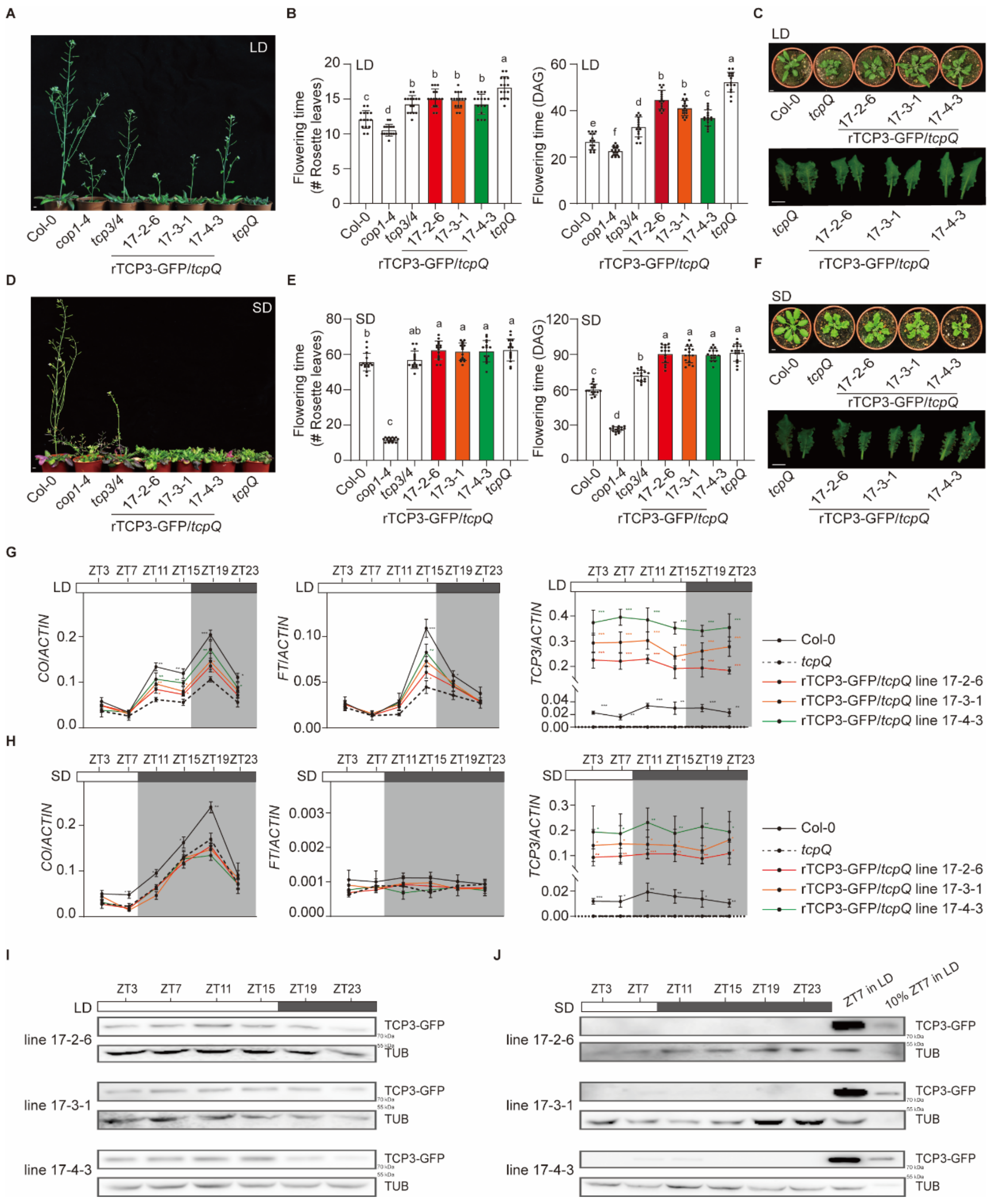

Fig S11: Influence of TCP3 on flowering time in long day (LD) and short day (SD). **(A-C) TCP3 accelerates flowering time in LD.** Visual phenotypes of specified genotypes including three independent transgenic *rTCP3-GFP/tcpQ* lines cultivated in LD for 6 weeks **(A)** or 3 weeks **(C)**. Scale bar=1 cm. Flowering time was quantified **(B)** as rosette leaf numbers and days after germination (DAG) at bolting time. Error bars represent the standard deviation of 15 plants. **(D-F) TCP3 has a weak effect on flowering time in SD.** Visual phenotype of specified genotypes cultivated in SD for 12 weeks **(D)** or 4 weeks **(F)**. Scale bar=1 cm. Flowering time was quantified **(E)** as rosette leaf numbers and DAG. Error bars represent the standard deviation of 15 plants. Letters in **B** and **E** denote significant differences between genotypes; genotypes sharing the same letter do not significantly differ, as determined by one-way analysis of variance (ANOVA) ($p < 0.05$). **(G, H) Transcript levels of *CO*, *FT* and *TCP3* at indicated Zeitgeber Times (ZTs) in LD and**

SD. rTCP3-GFP/*tcpQ* plants were grown in LD for 3 weeks **(C)** or SD for 7 weeks **(F)**. Leaves were sampled every 4 h at indicated ZTs. Different plant ages were used due to the slower plant growth in SD than in LD; plants were approximately at the same developmental stage in LD and SD. Transcript levels were normalized to *ACTIN*. Error bars represent the standard deviation from three biological replicates. Asterisks denote significant differences of transgenic lines compared to *tcpQ* (Student's *t* test, ***$p < 0.001$, **$p < 0.01$, *$p < 0.05$).

(I, J) Protein levels of TCP3-GFP at indicated ZTs in LD (I) and SD (J). Aliquots of the same frozen plant material sampled in panels G and H were used for protein extraction. In panel **J**, protein extract of LD-grown plants at ZT7 was used for comparison (10% refers to a 1:10 dilution of the extract). TCP3-GFP and TUB proteins were detected using α-GFP and anti-α-tubulin antibodies, respectively.

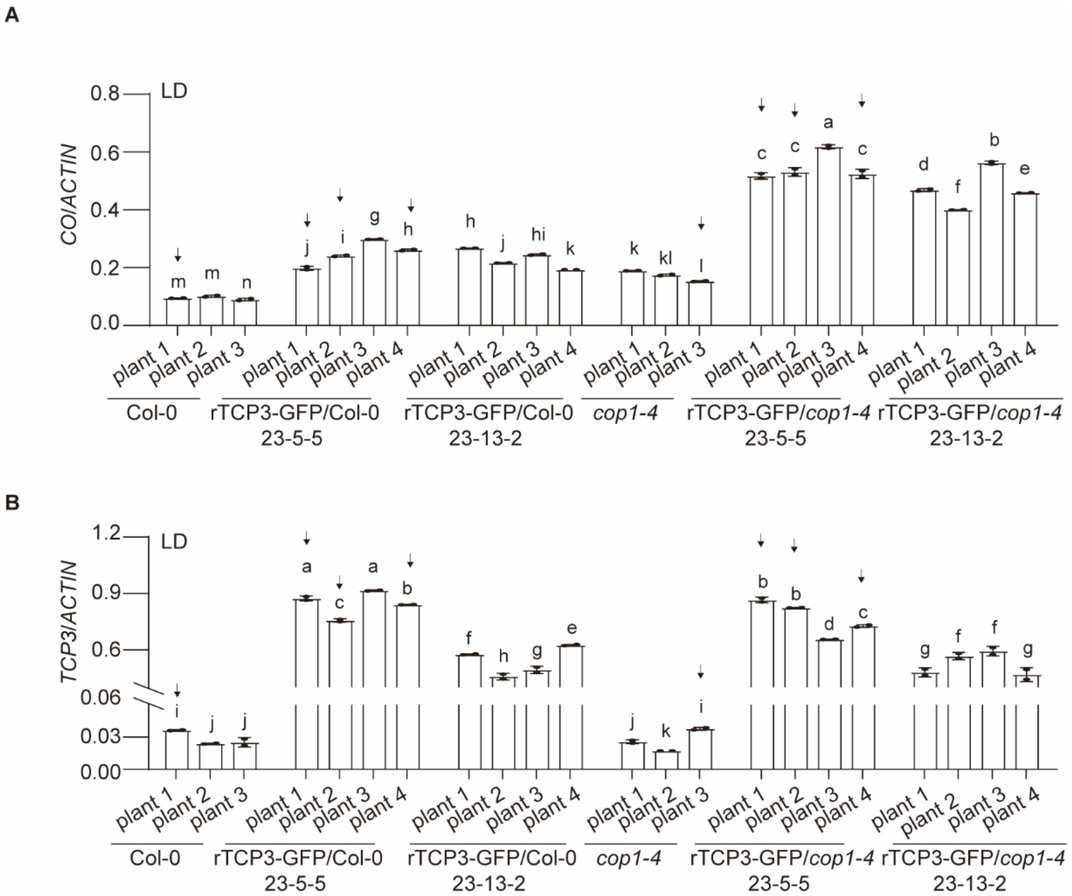

Fig S12: Transcript levels of *CO* and *TCP*3 in rTCP-GFP/Col-0 or rTCP-GFP/*cop1-4* plants in LD.

(A, B) Transcript levels of *CO* **(A)** and *TCP3* **(B)** were analyzed in individual plants of the indicated genotypes grown in LD for 3 weeks in order to assess plant-to-plant variability. Tissue was harvested at ZT15. Transcript levels were normalized to *ACTIN*. Error bars represent the standard deviation from two technical replicates. Letters denote significant differences between genotypes; genotypes sharing the same letter do not significantly differ, as determined by one-way ANOVA ($p < 0.05$).

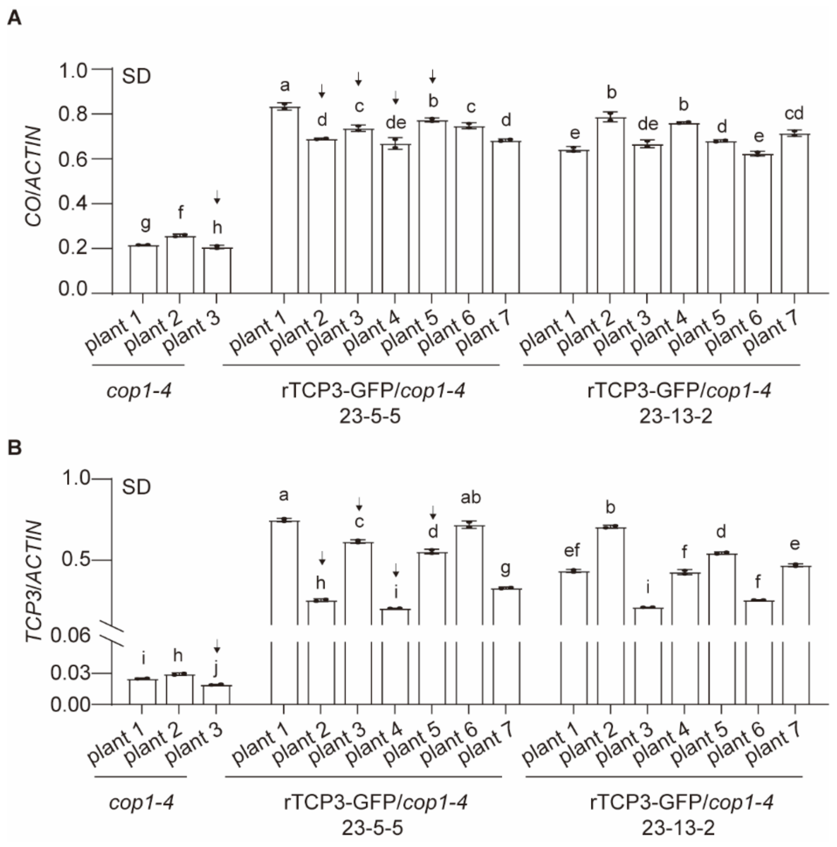

Fig S13: Transcript levels of *CO* and *TCP3* transcript levels in rTCP3-GFP/*cop1-4* plants in SD.

(A, B) Transcript levels of *CO* **(A)** and *TCP3* **(B)** were analyzed in individual plants of rTCP3-GFP/*cop1-4* (line 23-5-5/*cop1-4* and line 23-13-2/*cop1-4*) grown in SD for 3 weeks in order to assess plant-to-plant variability. Tissue was harvested at ZT7. Transcript levels were normalized to *ACTIN*. Error bars represent the standard deviation from two technical replicates. Genotypes sharing the same letter do not significantly differ, as determined by one-way ANOVA ($p < 0.05$). The transcript levels of *TCP3* and *CO* in Col-0 and rTCP3-GFP /Col-0 (line 23-5-5 and 23-13-2) are shown in **S14E** and **S14F**.

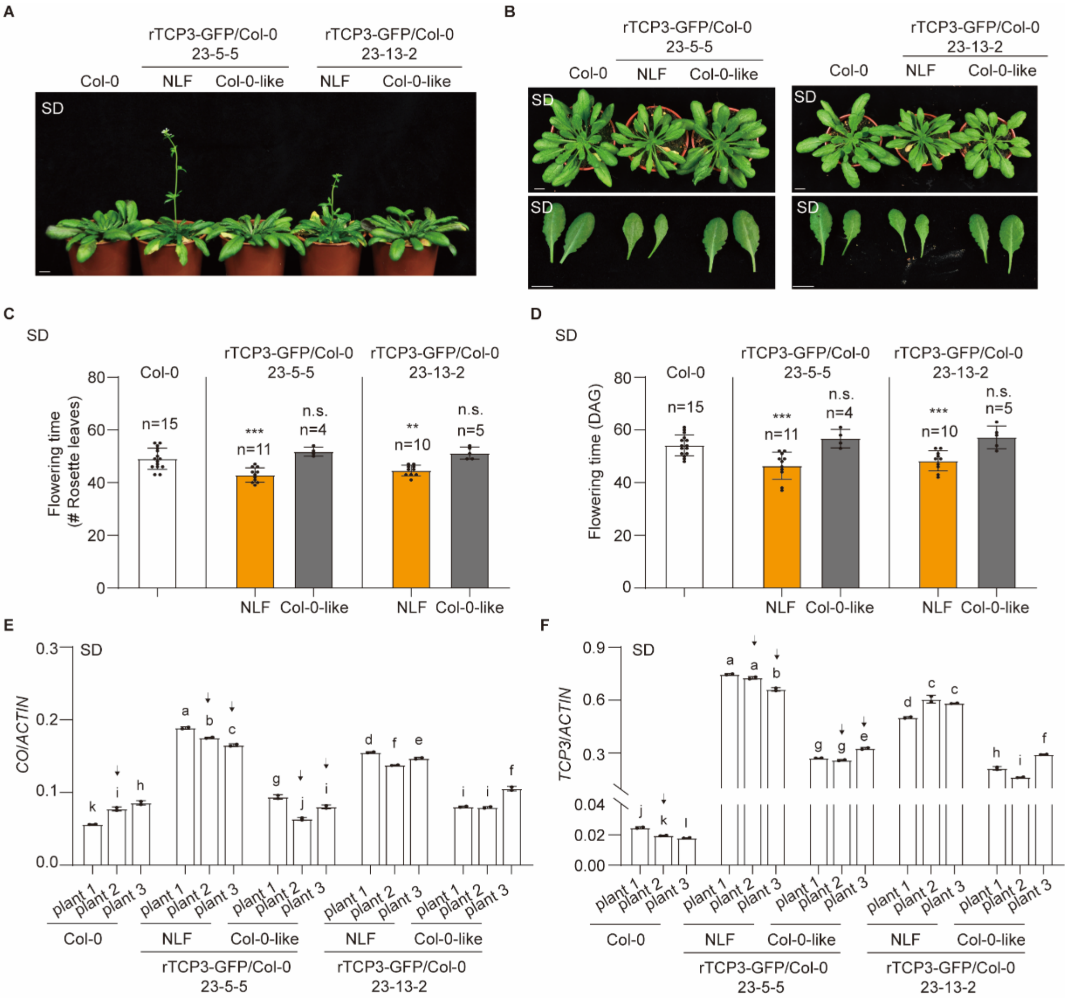

Fig S14: Effects of TCP3-GFP over-expression in a Col-0 wild-type background on flowering time and *CO* transcript levels in plants grown in SD

(A, B) Visual phenotype of representative Col-0 and rTCP3-GFP/Col-0 plants grown in SD for 8 weeks **(A)** or 5 weeks **(B)**. If the leaf shape is not different from Col-0, it is classified as Col-0-like. If plants have a narrow leaf shape (NLF) phenotype, it is classified as NLF. Scale bar= 1 cm

(C, D) Quantification of flowering time of plants grown in SD. Days after germination (DAG) and rosette leaf numbers at bolting time were analyzed separately for plants showing the NLF or Col-0-like leaf phenotype (n=15). The error bars represent the standard deviation. Asterisks denote significant differences of crossing lines compared to *cop1-4* (Student's *t* test, ***$p < 0.001$, **$p < 0.01$).

(E-F) Transcript levels of *CO* (E) and *TCP3* (F) in individual plants of the indicated genotypes grown in SD. Individual plants of the indicated genotypes were grown in SD for 7 weeks in order to assess plant-to-plant variability. Tissue was harvested at ZT7. Transcript levels were normalized to *ACTIN*. Error bars represent the standard deviation from two technical replicates. Genotypes sharing the same letter do not significantly differ, as determined by one-way ANOVA ($p < 0.05$). The transcript levels of *CO* and *TCP3* in *cop1-4* and rTCP3-GFP /*cop1-4* are shown in **S13A** and **S13B**.

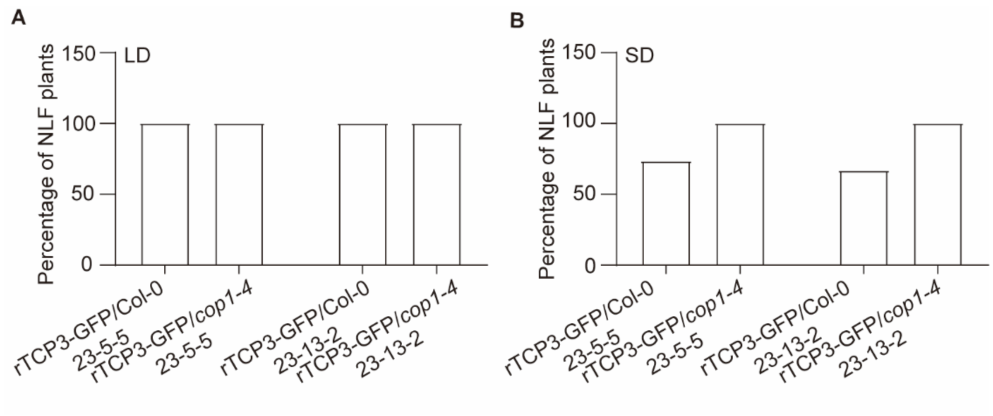

Fig S15: Comparison of the proportion of plants showing a Col-0-like and a narrow-leaf like (NLF) phenotype in Col-0 wild-type and *cop1-4* mutant backgrounds.

The percentage of Col-0-like and NLF plants in Col-0 background and *cop1-4* background in LD **(A)** and SD **(B)**. 15 plants for each genotype were counted.

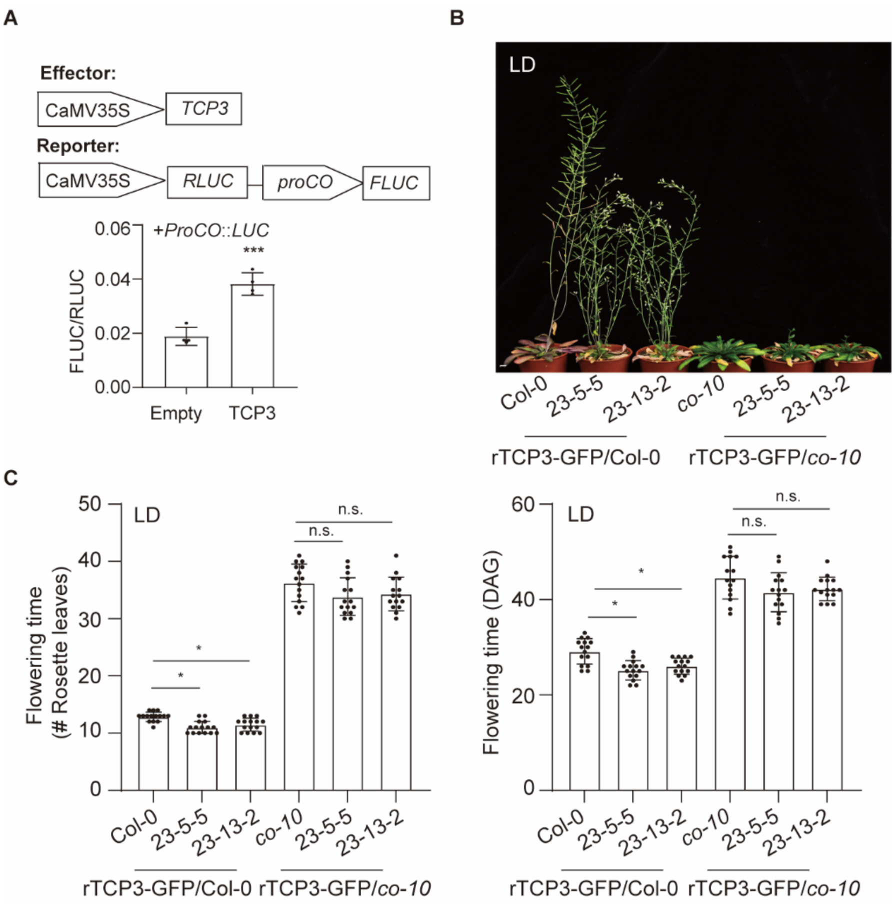

Fig S16: TCP3 action on flowering time in LD depends on *CO*

(A) TCP3 activates expression from *CO* promoter. The effector (*p35S*::*TCP3*) and reporter (*ProCO*::*FLUC*) constructs were co-infiltrated into tobacco leaves. FLUC and RLUC luminescence was quantified three days post-infiltration. Error bars indicate the standard deviation from four biological replicates (Student's *t* test, ***$p < 0.005$).

(B) Visual phenotype of representative plants of the indicated genotypes grown in LD for 8 weeks. Scale bar= 1 cm. The respective *rTCP3-GFP* transgene was transferred from the Col-0 wild-type into the *co-10* mutant background by crossing.

(C) Quantification of flowering time of the indicated genotypes grown in LD. Flowering time was quantified as days after germination (DAG) and rosette leaf numbers at bolting time. Error bars represent the standard deviation of 15 plants. Asterisks denote significant differences of crossing lines compared to Col-0 or *co-10* (Student's *t* test, *$p < 0.05$).

Table S1. Primer List

Purpose	Name	Primer sequence (5′>3′)	Orientation
Entry Cloning	TCP3-pEntry3c-F2	AACCAATTCAGTCGACTGGATCCATGGCACCAGATAACGACCATTTC	Forward
	TCP3-pEntry3c-R2	GAAAGCTGGGTCTAGATATCTCGAGCCATGGCGAGAATCGGATGAAGC	Reverse
	TCP3 MutJAW-R1	GATAGATTGCAGAGGACCCCT	Reverse
	TCP3 MutJAW-F1	AGGGGTCCTCTGCAATCTATC	Forward
	TCP4-pEntry3c-F2	AACCAATTCAGTCGACTGAAATGTCTGACGACCAATTCCATC	Forward
	TCP4- pEntry3c -R2	GAAAGCTGGGTCTAGATATCATGGCGAGAAATAGAGGAAGCAG	Reverse
	TCP10- pEntry3c -F1	AACCAATTCAGTCGACTGCGATGGGACTTAAAGGATATAGCGTCGG	Forward
	TCP10- pEntry3c -R2	GAAAGCTGGGTCTAGATATCTCGAGGAGGTGTGAGTTTGGAGGAGAAGAAGAAGAG	Reverse
	TCP3-D1-pEntry3c-F	AACCAATTCAGTCGACTGCGATGCCTAAGAAGAAGAGAAAGGTTATGGCACCAGATAACGACCATTT	Forward
	TCP3-D1-pEntry3c-R	GAAAGCTGGGTCTAGATATCGTTCCAAGGAGGAAGCTGAGCA	Reverse
	TCP3-D2-pEntry3c-F	AACCAATTCAGTCGACTGCGATGCCTAAGAAGAAGAGAAAGGTTCCCGCCGATACTCTTCGTCA	Forward
	TCP3-D2-pEntry3c- -R	GAAAGCTGGGTCTAGATATCATGGCGAGAATCGGATGAAGC	Reverse
	TCP3-D3-pEntry3c-F	AACCAATTCAGTCGACTGCGATGCCTAAGAAGAAGAGAAAGGTTAGAAAAGACAGACATAGTAAAGTATGTACAGCG	Forward
	TCP3-D3-pEntry3c-R	GAAAGCTGGGTCTAGATATCGTTCCAAGGAGGAAGCTGAGCA	Reverse
	TCP3-D4-pEntry3c-F	AACCAATTCAGTCGACTGCGATGCCTAAGAAGAAGAGAAAGGTTATACAGAGACTAGTGTCATGGAACAACG	Forward
	TCP3-D4-pEntry3c-R	GAAAGCTGGGTCTAGATATCATGGCGAGAATCGGATGAAGC	Reverse
	TCP10-AAmut-F2	CAGTCGTAGCCGCCGAGACCAACA	Forward

	TCP10-AAmut-R2	TGTTGGTCTCGGCGGCTACGACTG	Reverse
	NLS-GUS-Gib-fwd	CCAATTCAGTCGACTGGATCCATGCCTAAGAAGAAGAGAAAGGTTTTACGTCCTGTAGAA	Forward
	NLS-GUS-Gib-rev	GCTGGGTCTAGATATCTCGATGTTTGCCTCCCTGCTGCGG	Reverse
Y2H	COP1-pGBK-F	TATGGCCATGGAGGCCGAATTCATGAAGCTACTGTCTTCTATCGAACAAG	Forward
	COP1-pGBK-R	CGCTGCAGGTCGACGGATCCTCACGCAGCGAGTACCA	Reverse
	COP1-W467A-F	CGAAAAACGTGCCTGGAGTGTTGAC	Forward
	COP1-W467A-R	GTCAACACTCCAGGCACGTTTTTCG	Reverse
	COP1- F595A-F	CGAGAAGAACGCGGTGGGTCTCAC	Forward
	COP1- F595A-R	GTGAGACCCACCGCGTTCTTCTCG	Reverse
Y3H	COP1-vazyme-fwd	TGTATCGCCGGAATTGGATCACATGGAAGAGATTTCGACGGATCC	Forward
	COP1-vazyme-rev	GCTGCATTAACTAGTGAATTCTCACGCAGCGAGTACCAGAA	Reverse
	pBridge-BD-COP1+SPA1-F1	GAAGAAGAGAAAGGTGGCGGCCGCCATGCCTGTTATGGAAAGAGTAGC	Forward
	pBridge-BD-COP1+SPA1-R1	TCCTACCTAGGCTGCAGTCAAACAAGTTTTAGTAGCTTCATGTTTCCAGT	Reverse
	pBridge-BD-COP1+TCP10-F1	GAAGAAGAGAAAGGTGGCGGCCGATGGGACTTAAAGGATATAGCGTCG	Forward
	pBridge-BD-COP1+TCP10-R1	AACCAATTCAGTCGACTGCGATGGGACTTAAAGGATATAGCGTCGG	Reverse
LCI	TCP3-nLUC-F2	GGACGAGCTCGGTACCATGGCACCAGATAACGACCATTTCTTAGATTCT	Forward
	TCP3-nLUC-R2	GCGTACGAGATCTGGTCGACATGGCGAGAATCGGATGAAGC	Reverse
	KpnI-COP1-fwd	CGCAGCGGTACCATGGAAGAGATTTCGACGGATCC	Forward
	SalI-Stop-COP1-	TGCAGGTCGACCTACGCAGCGAG	Reverse

	rev	TACCAGAAC	
	KpnI-SPA1-fwd	GCATCGGTACCATGCCTGTTATGG AAAGAG	Forward
	SalI-Stop-SPA1-rev	CTGCAGGTCGACCTAAACAAGTT TTAGTAGCTTCAT	Reverse
CRISPR-Cas9	TCP3-M1-F3	ATTGCAATCAGCGACGGAGAACG G	Forward
	TCP3-M1-R3	AAACCCGTTCTCCGTCGCTGATT G	Reverse
	TCP4-M2-F2	ATTGAATGAGGCACCGTTCTACG T	Forward
	TCP4-M2-R2	AAACACGTAGAACGGTGCCTCAT T	Reverse
	TCP10-M3-F3	ATTGAAGACCGGTTAGGTTATGA C	Forward
	TCP4-M3-R3	AAACGTCATAACCTAACCGGTCT T	Reverse
	TCP2-M4-F2	ATTGAGACTGATAACCGGACACG A	Forward
	TCP2-M4-R2	AAACTCGTGTCCGGTTATCAGTC T	Reverse
GST protein purification	TCP3-pGEX4T-1-F1	CGCGTGGATCCCCGGAAATGGCA CCAGATAACGACCATTT	Forward
	TCP3-pGEX4T-1-R1	TCACGATGCGGCCGCATGGCGAG AATCGGATGAAGC	Reverse
Dual-luciferase assays	TCP3-pGreen62-SK-F	TCTAGAACTAGTGGATCCATGGC ACCAGATAACGACCAT	Forward
	TCP3-pGreen62-SK-R	CGACGGTATCGATAAGCTTATGGC GAGAATCGGATGAA	Reverse
	PAP2-pGreen62-SK-F1	GCTCTAGAACTAGTGGATCCATG GAGGGTTCGTCCAAA	Forward
	PAP2-pGreen62-SK-R1	GGTCGACGGTATCGATAAGCTTC TAATCAAGTTCAACAGTCTCTCC ATCAAACAG	Reverse
	TT8-pGreen62-SK-F1	TCTAGAACTAGTGGATCCATGGAT GAATCAAGTATTATTCCGGC	Forward
	TT8-pGreen62-SK-R1	GGTCGACGGTATCGATAAGCTTC TATAGATTAGTATCATGTATTATGA CTTGGTGGATGG	Reverse
	pGreenII0800-DFRpro-F	GGTATCGATAAGCTTTTAAGTGAT TCACTGTCCTTCTAATTTTATTTTA TTTT	Forward
	pGreenII0800-DFRpro-R	TCTAGAACTAGTGGATCCTTTTGT GGTTATATGATAGATTGTGCTTTG	Reverse

		G	
	pGreenII0800-LDOX1pro-F	GGTCGACGGTATCGATAAGCTTGTGGAGATAAATTCCTAGGCAGATTTCTCT	Forward
	pGreenII0800-LDOX1pro-R	TAGAACTAGTGGATCCCTTCTTTAGTCTTCTGTTTAAAGCTAAAAC	Reverse
	proCO-LUC-F1	GGTCGACGGTATCGATAAGCTTAAGAGAAGTGCGGTGTAAGCAAATATGA	Forward
	proCO-LUC-R1	CCGCTCTAGAACTAGTGGATCCAATAACTCAGATGTAGTAAGTTTGATGGTGTGG	Reverse
RT-qPCR	Q-TCP3-F3	GGGATTCACCAATCTGCTATTCCA	Forward
	Q-TCP3-R3	TTAATGGCGAGAATCGGATGAAGC	Reverse
	Q-CO-F1	CAACAGCTTCACACCCAAGAACG	Forward
	Q-CO-R2	TTGCAGGGTCAGGTTGTTGCTC	Reverse
	Q-FT-F1	CCATTGGTTGGTGACTGATATCC	Forward
	Q-FT-R1	TTGCCAAAGGTTGTTCCAGTT	Reverse
	Q-CHS-F2	AGCTGATGGACCTGCAGGCATCTTGGC	Forward
	Q-CHS-R2	TGCATGTGACGTTTCCGAATTGTCGAC	Reverse
	Q-CHI-F2	CTTCGCTCTCTCCCCTACCG	Forward
	Q-CHI-R2	GATCACAGCGATCCCGGTTT	Reverse
	Q-DFR-F3	ACCGGAGATGGTTTAACCGATGGT	Forward
	Q-DFR-R3	TGGGAGCATCGGTTCTCTCGC	Reverse
	Q-LDOX-F2	TGGGTCACTGCAAAATGTGT	Forward
	Q-LDOX-R2	CGGAGACTCAACACTCACCA	Reverse
	Q-PAP1-F2	CTGGTCGGACCGCAAATGACGT	Forward
	Q-PAP1-R2	GGGCATTGAGATGGTTGCAGTCGT	Reverse
	Q-PAP2-F2	TGGGGGAAAACCAAGAAGCTGATGC	Forward
	Q-PAP2-R2	AACGTCAAACGCCAAAGTGGCC	Reverse
	Q-ACTIN-F	CAAGGCCGAGTATGATGAGG	Forward
	Q-ACTIN-R	GAAACGCAGACGTAAGTAAAAAC	Reverse

References:

1.X. Holtkotte, J. Ponnu, M. Ahmad, U. Hoecker, The blue light-induced interaction of cryptochrome 1 with COP1 requires SPA proteins during Arabidopsis light signaling. *PLoS Genet.* **13**, e1007044

(2017).
2.J. Ponnu, T. Riedel, E. Penner, A. Schrader, U. Hoecker, Cryptochrome 2 competes with COP1 substrates to repress COP1 ubiquitin ligase activity during Arabidopsis photomorphogenesis. *Proc. Natl. Acad. Sci. U.S.A.* **116**, 27133-27141 (2019).
3.A. Steffens, B. Jaegle, A. Tresch, M. Hülskamp, M. Jakoby, Processing-Body Movement in Arabidopsis Depends on an Interaction between Myosins and DECAPPING PROTEIN1. *Plant Physiol.* **164**, 1879-1892 (2014).
4.S. Laubinger, K. Fittinghoff, U. Hoecker, The SPA Quartet: A Family of WD-Repeat Proteins with a Central Role in Suppression of Photomorphogenesis in Arabidopsis. *Plant cell* **16**, 2293-2306 (2004).
5.N. Ordoñez-Herrera *et al.*, The transcription factor COL12 is a substrate of the COP1/SPA E3 ligase and regulates flowering time and plant architecture. *Plant Physiol.* **176**, 1327-1340 (2017).
6.H. Chen *et al.*, Firefly Luciferase Complementation Imaging Assay for Protein-Protein Interactions in Plants. *Plant Physiol.* **146**, 323-324 (2007).
7.T. L. Shimada, T. Shimada, I. Hara-Nishimura, A rapid and non-destructive screenable marker, FAST, for identifying transformed seeds of Arabidopsis thaliana. **61**, 519-528 (2010).
8.R. P. Hellens *et al.*, Transient expression vectors for functional genomics, quantification of promoter activity and RNA silencing in plants. *Plant methods* **1**, 13 (2005).
9.J. Ordon *et al.*, Generation of chromosomal deletions in dicotyledonous plants employing a user-friendly genome editing toolkit. *Plant J* **89**, 155-168 (2017).
10.M. Kreiss, F. B. Haas, M. Hansen, S. A. Rensing, U. Hoecker, Co-action of COP1, SPA and cryptochrome in light signal transduction and photomorphogenesis of the moss Physcomitrium patens. **114**, 159-175 (2023).
